# Niche partitioning of the ubiquitous and ecologically relevant NS5 marine group

**DOI:** 10.1038/s41396-022-01209-8

**Published:** 2022-02-15

**Authors:** Taylor Priest, Anneke Heins, Jens Harder, Rudolf Amann, Bernhard M. Fuchs

**Affiliations:** Department of Molecular Ecology, Max Planck Institute for Marine Microbiology, Bremen, Germany

**Keywords:** Microbial ecology, Marine microbiology, Water microbiology

## Abstract

Niche concept is a core tenet of ecology that has recently been applied in marine microbial research to describe the partitioning of taxa based either on adaptations to specific conditions across environments or on adaptations to specialised substrates. In this study, we combine spatiotemporal dynamics and predicted substrate utilisation to describe species-level niche partitioning within the NS5 Marine Group. Despite NS5 representing one of the most abundant marine flavobacterial clades from across the world’s oceans, our knowledge on their phylogenetic diversity and ecological functions is limited. Using novel and database-derived 16S rRNA gene and ribosomal protein sequences, we delineate the NS5 into 35 distinct species-level clusters, contained within four novel candidate genera. One candidate species, “*Arcticimaribacter forsetii* AHE01FL”, includes a novel cultured isolate, for which we provide a complete genome sequence—the first of an NS5—along with morphological insights using transmission electron microscopy. Assessing species’ spatial distribution dynamics across the Tara Oceans dataset, we identify depth as a key influencing factor, with 32 species preferring surface waters, as well as distinct patterns in relation to temperature, oxygen and salinity. Each species harbours a unique substrate-degradation potential along with predicted substrates conserved at the genus-level, e.g. alginate in NS5_F. Successional dynamics were observed for three species in a time-series dataset, likely driven by specialised substrate adaptations. We propose that the ecological niche partitioning of NS5 species is mainly based on specific abiotic factors, which define the niche space, and substrate availability that drive the species-specific temporal dynamics.

## Introduction

An ecological niche is defined as a specific set of conditions (environmental and biotic interactions) that allow a population to perform its evolutionarily adapted function and as a result, persist or grow [1, 2]. Although a long-standing concept in ecology, niche theory has only recently been incorporated in the study of marine microbial populations [3–6]. Such studies have either focused on adaptations to specific conditions across environments [4] or on specific functional adaptations within an environment, e.g. specialised substrate utilisation [7]. However, to obtain a more detailed understanding on microbial populations’ niches, an in-depth analysis on the adaptation to conditions across different spatial and temporal scales in combination with an assessment of ecological function is needed.

Microbial populations exhibit distinct distribution patterns across the world’s oceans, which are most influenced by depth [8] and changes in temperature [9, 10] and salinity [11]. However, the effect these have on microbial populations varies. Although some appear to be ubiquitously distributed, such as the SAR11 or *Prochlorococcus* Clade, further analysis has shown that distinct genetic variations exist, resulting in ecotypes that are driven by environmentally mediated selection processes [9, 12]. Within specific environments, microbial populations also exhibit distinct dynamics that are driven by temporally derived shifts, such as seasons [6]. This is particularly evident with heterotrophic microbes in the *Bacteroidetes* phylum, that show recurrent and potentially predictable, seasonal dynamics driven by substrate availability [7, 13, 14]. From these studies, it is clear that conditions and resources influence microbial populations, however, to what extent do these determine niches?

In this study, we phylogenetically and ecologically characterise members of the NS5 marine group (referred to as NS5 from hereon) and subsequently identify the key niche-determining factors over spatial and temporal scales. The NS5 was selected as it represents a ubiquitous and abundant group of the *Flavobacteriia* class for which our knowledge on phylogeny and function is limited. Since the name was introduced 14 years ago, from a study describing high local and temporal diversity of *Flavobacteriia* in the North Sea [15], NS5-classified sequences have been recovered from across the world’s marine water masses, ranging from semi-enclosed seas in tropical regions [16, 17] to the Antarctic peninsula [18] and North Pacific oxygen minimum zone [19]. They are frequently reported as one of the most abundant groups of *Flavobacteriia* from studies using 16S rRNA gene analysis [17, 20] and fluorescence in situ hybridisation (FISH) cell counts [21] (referred to as VIS1 in that study). The VIS1 clade, which represents only a fraction of the NS5, was reported to reach 29 ± 3 × 10^3^ cells ml^−1^ in the Arctic province of the North Atlantic. Members of the NS5 have been shown to associate with spring phytoplankton blooms [13, 22, 23] and increasing chlorophyll *α* concentrations [21, 24], however they are typically more prominent in early bloom stages or are more tightly coupled to the fluctuations in flagellate abundance [13]. A study conducted in the South Sea of Korea concluded that NS5 was a good indicator species for coastal waters [25] whilst they were also shown to be linked to eutrophication in coastal bays of Vietnam [22]. In contrast, other findings have indicated NS5 sequence abundance maxima to occur in winter [26] and a dominance of NS5 affiliated sequences in open ocean Arctic waters [20, 21], which motivated us to here provide novel data from the Fram Strait region. From these studies, it is clear that the NS5 may represent a diverse group of bacteria with different ecological niches.

With a global perspective, we here characterise the NS5 based on (1) phylogenetic analysis using 16S rRNA gene and ribosomal protein tree reconstructions, (2) functional descriptions using MAGs and a complete genome of a cultured isolate, (3) spatiotemporal distribution patterns of species-level cluster representatives and (4) visual identification of cells in culture using transmission electron microscopy (TEM) and in the environment using catalysed reporter deposition-fluorescence in situ hybridisation (CARD-FISH). As a result, we identify and characterise 35 species assigned to four novel candidate genera, which we have named *Candidatus* Marisimplicoccus (NS5_A), *Candidatus* Marivariicella (NS5_B), *Candidatus* Maricapacicella (NS5_D) and *Candidatus* Arcticimaribacter (NS5_F), for each of which, we propose a candidate type species based on a genome voucher.

## Materials and methods

### Fram Strait sampling and sequencing

Seawater samples were collected at the deep chlorophyll maximum (DCM) layer from 11 stations across the Fram Strait region in July and August 2018 during the PS114 Polarstern cruise, as described previously [27]. Seawater was fractionated using filtration and DNA extracted using a modified SDS-based extraction method after Zhou et al. [28]. Metagenomes were generated from the 0.2–3 µm fraction using the HiSeq 3000 (Illumina) and Sequel II (PacBio) platforms, as described previously [27] (Supplementary Material 1).

### Generation of MAG dataset

The metagenomic reads from the Fram Strait samples were assembled (using Megahit [29] for Illumina reads and MetaFlye [30] for PacBio reads), binned (using Concoct [31], Metabat2 [32], Maxbin2 [33] and DAStool [34]) and manually refined as described previously [27], resulting in MAGs identified in this study by the prefix “FRAM18_“. Estimation of genome completion and contamination was determined using CheckM v1.1.2 [35]. MAGs belonging to the NS5 were identified by 16S rRNA gene phylogeny (Supplementary Material 1) and additionally assigned to taxonomic groups in the Genome Taxonomy Database (GTDB) (Release 89) using the classify_wf pipeline of GTDB-tk v1.0.2 [36, 37]. The dataset was expanded by retrieving all species-representative assemblies within the assigned GTDB taxa along with MAGs from two additional 0.2–3 µm marine microbial metagenomic datasets (Bioproject accessions: PRJEB28156 [38, 39], PRJEB43746) that had been assigned the same GTDB taxonomy (Supplementary Tables S1 and S2). The resulting dataset was de-replicated using FastANI v1.9 [40] with a cut-off threshold of 95%.

### Helgoland NS5 isolate AHE01FL: sampling, isolation and genome sequencing

Seawater from the long-term ecological research station Helgoland Roads (54°11′03″N, 7°54′00″E) was sampled on the 28th April, 2016 and serially diluted with artificial seawater [41]. An inoculum of 2.6 nl, statistically containing three cells, was grown in an oligotrophic HaHa medium with the addition of vitamins [42] in the dark at 12 °C. After several transfers and another dilution to extinction series, purity controls confirmed that the culture contained a pure strain. It was maintained by transfers every 3 months. Growth in HaHa100V medium [42] yielded a turbid, orange-coloured culture and provided biomass for DNA extraction that was performed according to Zhou et al. [28]. Genome sequencing was performed by the Max Planck-Genome-centre Cologne, Germany (https://mpgc.mpipz.mpg.de/home/) using Sequel I (PacBio) and HiSeq 2500 (Illumina) platforms. Circular long read sequences from PacBio were assembled using Canu v2.1 [43] whilst short Illumina reads were assembled using Spades v3.13.2 (parameters: -isolate) [44]. The contigs from both datasets were aligned and assembled together in Geneious Prime v2019.1.3 (https://www.geneious.com) before a final round of error-correction using the Illumina reads as a reference. The assembled genome was submitted to EMBL-EBI and assigned the name “*Flavobacteriaceae* bacterium AHE01FL” with the taxid 2820661 and in this study, is named “Iso_AHE01FL”.

### Helgoland NS5 isolate AHE01FL: cell visualisation

To accurately determine cell morphology of Iso_AHE01FL, TEM was used. An aliquot of the liquid culture was retrieved and fixed with 25% glutaraldehyde (EM grade Science Services) for 1 h at room temperature followed by centrifugation (5 min at 21,100 × *g*) and resuspension in the growth media (HaHa 100 V medium). An aliquot of this resuspension was pipetted onto a Formvar coated 400-mesh copper grid and stained with 1% uranylacetate for 5 min before being air dried overnight.

### NS5 MAG phylogenetic tree reconstruction

The reconstruction of a phylogenetic tree for species-representative MAGs was performed using a concatenated alignment of 16 ribosomal proteins (L2, L3, L4, L5, L6, L14, L16, L18, L22, L24, S3, S8, S10, S17, S19), following the procedure described by Hug et al. [45]. In brief, Muscle v3.8.15 [46] was used to align amino acid sequences that were subsequently trimmed using TrimAI v1.4.1 [47] and concatenated into a single alignment that was provided as an input to FastTree v2.1.10 [48] (Supplementary Material S1). This workflow was then repeated with the addition of 1275 *Flavobacteriaceae* assemblies from the RefSeq database (Supplementary Material S1). To corroborate the inferred phylogenetic separation of MAGs, average nucleotide identity (ANI) and average amino acid identity (AAI) were calculated using FastANI and CompareM v0.1.1 (https://github.com/dparks1134/CompareM), respectively.

### 16S rRNA phylogenetic tree construction

16S rRNA gene sequences, longer than 1 kbp in length, were extracted from species-representative MAGs using Barrnap [49]. The sequences were imported to the ARB programme [50], aligned using the SINA aligner [51] and phylogenetically placed into the SILVA 138.1 SSU Ref NR99 reference tree using the parsimony algorithm. The MAG-derived sequences along with 100 of the highest quality NS5 sequences in the SILVA database were used for phylogenetic tree reconstruction. Three tree algorithms were used, RaxML v8.2.8 maximum likelihood (GTR-Gamma rate distribution model, rapid bootstrap algorithm, 100 repetitions) [52], neighbour-joining (Jukes-Cantor’s substitution model, 1000 bootstrap repetitions) and Parsimony v3.6, each with two different positional variability conservation filters, a 30% for all *Flavobacteriia* and the “termini” filter provided with the ARB SILVA database. A consensus tree was constructed from these six input trees and groups that remained stable throughout all tree methods were designated.

### Probe design and environmental cell visualisation

CARD-FISH [53, 54] probes could be designed in ARB for two genus-level clades, NS5_A and NS5_F (Supplementary Table S2). Optimal hybridisation conditions were determined by testing on filtered pelagic water samples from the Fram Strait region [27], the same samples used to generate the “FRAM18_” MAGs. The probes were subsequently applied to five samples from that dataset to obtain information on morphology and cell count data. More detailed information is provided in Supplementary Material S1.

### Global distribution of NS5 subgroups, MAGs and their correlation to physical parameters

The distribution of NS5 members was determined by recruiting metagenomic reads from the Tara Oceans dataset (ENA study accessions: PRJEB1787, PRJEB9740) [55] to species-representative MAGs using BBMap v38.73 [56], with a 99% identity threshold (minid = 99, idfilter = 99). In total, 122 surface water, 95 DCM and 47 mesopelagic metagenomes were used. To provide comparability between samples, the number of mapped reads was converted to reads per kilobase per million (RPKM) [57]. The generated data were imported into RStudio [58] and visualised using the packages *rnaturalearth* [59]*, sf* [60] and *ggplot2* [61]. To check the accuracy and provide support for the RPKM values, comparisons were made to genome coverage of mapped reads, cell counts (see “Probe design and environmental cell visualisation”) and another, more robust metric, the truncated average depth (TAD) [62], detailed information is provided in Supplementary Material S1.

To determine the effect of abiotic characteristics on NS5 species distribution, physical parameter measurements (depth, chlorophyll *a*, nitrite, nitrate + nitrite, oxygen, phosphate, salinity and silicate) of Tara Oceans samples were obtained from ENA-EBI. Scatter plots of RPKM values across physical parameters were produced using ggplot2 and Pearson’s correlation analysis performed using log transformed parameter values.

### Seasonal dynamics of species-representative MAGs

Temporal dynamics of species-representative MAGs was determined by read recruitment of oligotypes from a multiyear time-series dataset [63] sampled at Helgoland Roads, North Sea (Supplementary Material S1). Recruitment was performed by BBMap with a 100% identity threshold (minid = 100, idfilter = 100). The distribution dynamics, based on relative abundance of oligotypes taken from the original manuscript, were visualised using the *vegan* [64] and *ggplot2* packages in RStudio.

### Functional characterisation

The presence of major metabolic pathways was determined using KofamKoala [65] and RAST v2.0 [66]. For each MAG, initial gene prediction was performed by Prokka v1.14.6 [67]. Carbohydrate-active enzymes (CAZymes) were predicted using a combination of HMMscan against the dbCAN v9 database [68] (*E*-value threshold: 1E−5) and Diamond blastp v0.9.14 [69] against the CAZy database (release 07312020) [70] (*E*-value threshold: 1E−20, parameters: -more-sensitive –query-cover 40 –id 30 –k 15). Sulfatases were annotated by blastp search against the SulfAtlas v1.3 database [71] (*E*-value threshold: 1E−4) and HMMscan against the Pfam sulfatase family PF00884 (*E*-value threshold: 1E−5). Peptidases were identified by blastp search against the MEROPS database [72] (*E*-value threshold: 1E−4). TonB-dependent transporters (TBDTs) were predicted by HMMscan against TIGRFAM profiles TIGR01352, TIGR01776, TIGR01778, TIGR01779, TIGR01782, TIGR01783, TIGR01785, TIGR01786, TIGR02796, TIGR02797, TIGR02803, TIGR02804, TIGR02805, TIGR04056 and TIGR04057 (*E*-value threshold: 1E−10). SusD genes were identified by HMMscan against the Pfam profiles PF12741, PF12771, PF14322, PF07980. Annotations of carbohydrate esterases, carbohydrate binding modules, glycoside hydrolases (GH) and polysaccharide lyases (PL) were designated correct only if both the dbCAN and CAZy annotations agreed. Annotations were combined into a single “gene_table” for each MAG. To identify potential polysaccharide utilisation loci (PULs), text searches were performed in the “gene_table” for regions on contigs that contained either a SusC/SusD gene pair with two or more degradative CAZymes or contained at least three substrate utilisation genes in close proximity (maximum of 6 genes in between each). PULs were manually inspected and visualised using the *gggenes* [73] and *ggplot2* packages in RStudio.

The composition of CAZyme, sulfatase, peptidase and TBDT gene families for all MAGs was subsequently converted to a Bray-Curtis dissimilarity matrix and used as an input for hierarchical clustering and a non-metric multi-dimensional scaling analysis, using the hclust and metaMDS functions of the *vegan* package in RStudio. The visualisation of the analyses was carried out using the *ggplot2* and *ggdendro* [74] packages.

### SusC/SusD protein trees

Amino acid sequences of SusC/SusD genes identified in PULs were extracted and used for tree calculation. Additional SusC/SusD sequences were included from previously published marine flavobacteria MAGs [38] and cultured isolates [75]. Multiple sequence alignments were calculated using MAFFT v7.310 [76] with L-INS-I and trees calculated using FastTree. Trees were visualised and annotated in iTOL v4 [77].

## Results

Seven species-representative MAGs retrieved from Fram Strait metagenomes were identified as members of the NS5 marine group through 16S rRNA gene analysis and assigned to four different genera within the GTDB database (MED-G11, GCA-002723295, MS024-2A, UBA7428). The GTDB species-representatives within these groups along with MAGs from two other metagenome datasets were acquired (Supplementary Tables S1 and S2). In addition, we sequenced and assembled a complete genome of an isolate retrieved from surface seawater at Helgoland Roads, North Sea in 2016. The derived dataset of assembled genomes was de-replicated at a 95% ANI threshold, resulting in 35 species-level clusters that provided the foundation for a detailed phylogenetic and ecological characterisation of the NS5 marine group (Supplementary Table S1).

### Phylogenetic analysis of the NS5 marine group

**Fig. 1:**
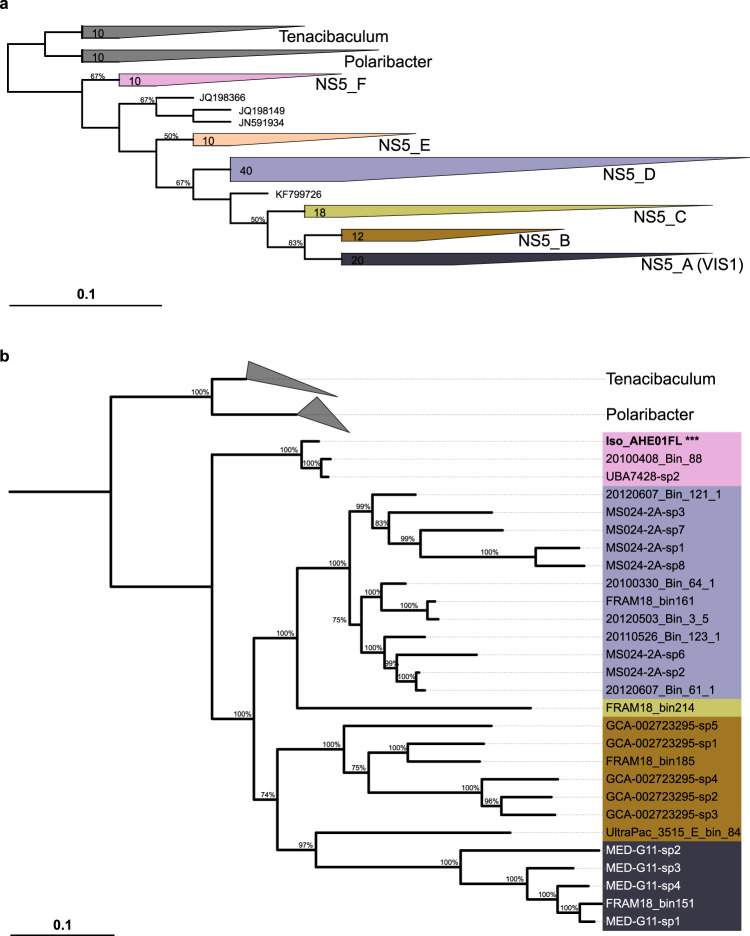
Phylogenetic tree reconstruction of the NS5 Marine Group. **a** 16S rRNA gene tree constructed using MAG sequences, from this and previous studies and the GTDB database, and 100 sequences classified as NS5 Marine Group in the SILVA 138 database. The tree represents a consensus from six input trees, constructed using three different algorithms, RaxML, Neighbour-joining and parsimony, with two different positional variability filters. **b** Ribosomal protein tree generated from a concatenated alignment of 16 proteins identified within NS5 species-representatives MAGs and genomes of Polaribacter and Tenacibaculum retrieved from the NCBI RefSeq database. The cultured isolate, Iso_AHE01FL, is highlighted in bold and with ***.

Phylogenetic tree reconstruction using 16S rRNA genes from NS5 species-representatives and sequences from the SILVA 138 database resulted in six distinct clusters being formed (NS5_A–NS5_F) (Fig. 1a). However, the MAG sequences were positioned only within five of the ribosomal protein-based clusters (not NS5_E), indicating that part of NS5’s diversity is not yet captured by MAGs. Minimum intra-group 16S rRNA gene sequence similarity varied from 93% in NS5_D to 97.0% in NS5_F whilst the median values ranged from 94.5% in NS5_B to 98.9% in NS5_F. The lower values observed were typically a result of only a few sequences, with the majority of minimum values being >94.5% and median values >96.4% and therefore, in agreement with genus-level thresholds [78].

Reconstruction of a MAG-based ribosomal protein tree (Fig. 1b) revealed five distinct groups that corresponded to clusters in the 16S rRNA gene tree. The number of species-representative MAGs in each cluster ranged from 17 in NS5_D to 1 in NS5_C. Genomic comparisons between MAGs revealed intra-cluster average AAI values of >65% and inter-cluster values of <65%, further supporting the delineation of groups at the genus-level [79]. The coherence and stability of the clusters was additionally confirmed by phylogenetic tree reconstruction at the family level (Supplementary Fig. S1). Due to the genetic coherence, the defined clusters will now be referred to as genera. The cultured isolate, Iso_AHE01FL, belonged to the NS5_F genus. In order to provide an indication on genetic conservation, the species-representative genomes from NS5_F were aligned to the complete isolate genome and a visualisation provided on the conserved syntenic gene blocks identified (Supplementary Fig. S2).

Clear distinctions between genera were evident with respect to genome size and GC content (Supplementary Fig. S3). The average genome size of the three most complete MAGs from each genus were 2.05 Mbp for NS5_F, 2.02 Mbp for NS5_D, 1.82 Mbp for NS5_B and 1.17 Mbp for NS5_A, whilst the GC content of NS5_A and _B representatives was ~30% compared to 36 – 37% in NS5_D and _F.

### Cell visualisation

**Fig. 2:**
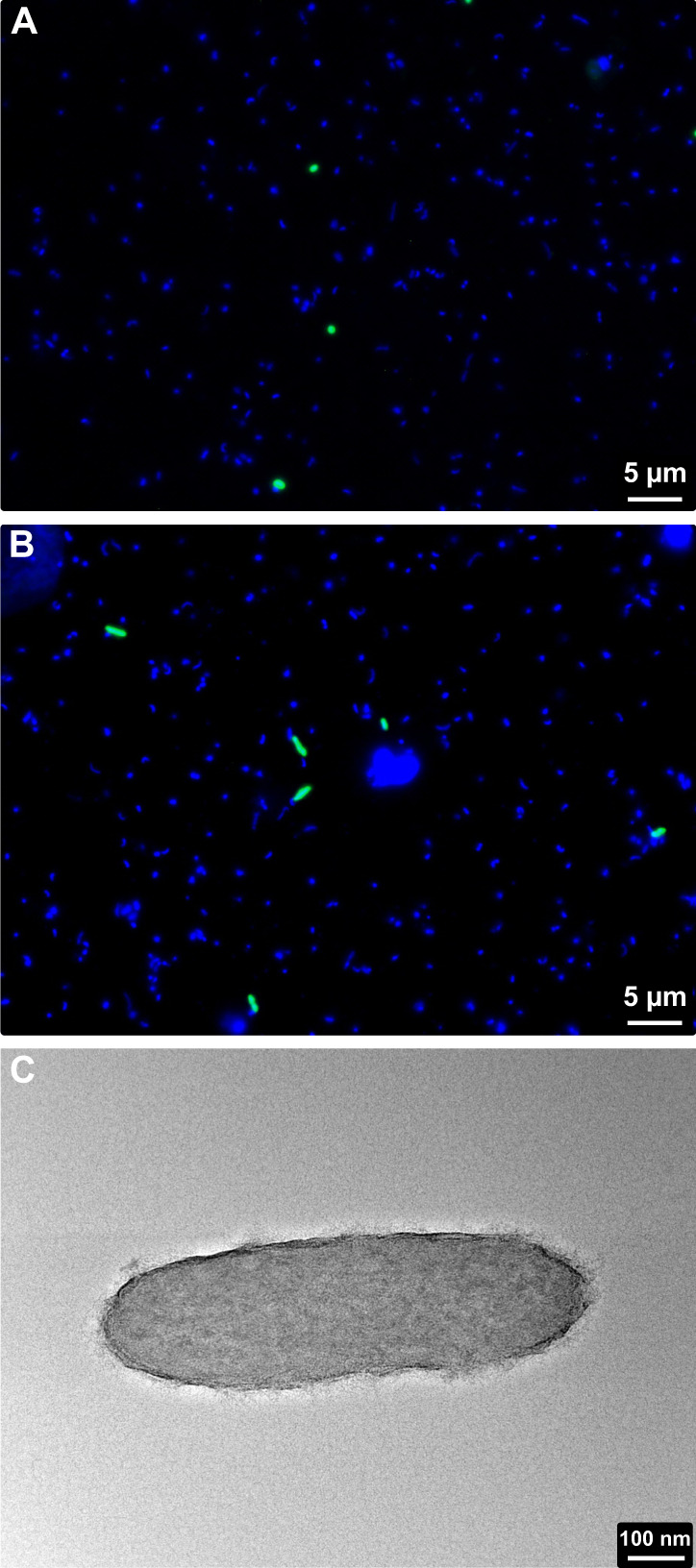
Visualisation of cells from NS5_A and NS5_F. Environmental cells hybridised using CARD-FISH probes targeting the NS5_A (**A**) and NS5_F (**B**). FISH probe signals are shown in green and DNA stain in blue. **C** Transmission electron microscopy image of the isolate, Iso_AHE01FL, in the NS5_F.

Following the design and optimisation of CARD-FISH probes (Supplementary Material S1) for the NS5_A and NS5_F genera (Supplementary Table S3), cells were visualised on filtered seawater samples from the Fram Strait region [27] (Fig. 2). Probe design for the other genera was unsuccessful due to sequence similarities with neighbouring taxa. Hybridised cells visualised using the NS5_A probe were of a small coccoid shape with a diameter of ~0.5 µm. Those identified with the NS5_F probe were rod-shaped cells with a length of 0.5–1.5 µm and width of ~0.5 µm. Enumeration of FISH signals that overlapped with a nucleic acid stain (DAPI) revealed similar peak counts for both NS5_A, 1.70 × 10^4^ cells ml^−1^, and NS5_F, 1.76 × 10^4^ cells ml^−1^ (Supplementary Fig. S4).

TEM on the Iso_AHE01FL revealed rod-shaped cells with a length of 0.5–1 µm and width of <0.5 µm (Fig. 2), in agreement with the observations on environmental samples, based on FISH.

### Global distribution of NS5 genera, MAGs and their correlation to physical parameters

The distribution of NS5 genera was determined by read recruitment from Tara Oceans metagenomes to each individual species and subsequently summing the RPKM values (Supplementary Fig. S5). To provide additional support, RPKM values were compared to genome coverage of mapped reads, CARD-FISH cell counts and another, more robust sequence-based metric, the TAD [62] (Supplementary Material S1 and Supplementary Figs. S4, S6 and S7). Based on this, a cut-off threshold of 0.25 RPKM was applied for inclusion in further analysis, which ensured a coverage of >40%. The four genera each exhibited a ubiquitous presence across all oceanic regions in the surface and DCM layers, although the NS5_F genus was less widespread in the DCM than surface. All genera showed lower RPKM values in the mesopelagic than DCM layer. The magnitude of RPKM values observed for NS5_B was six-fold lower than for the other genera. Variations in distribution patterns were evident, with the NS5_D and NS5_F reaching higher RPKM values in Arctic and geographically connected areas whilst NS5_A appeared more prevalent in specific locations, such as the North Atlantic and Chilean upwelling system. These patterns were further confirmed by grouping samples into oceanic regions (Supplementary Fig. S8).

On a species-level, an almost universal distribution pattern with depth was identified, with all but two species exhibiting highest RPKM values in surface waters (<30 m) (Supplementary Fig. S9). The two contrasting species were MS024-2A_sp7 (NS5_D), which peaked between 100–200 m, and MED-G11_sp2 (NS5_A), which peaked at ~300 m depth. Additionally, several species exhibited a bimodal peak, with highest values in surface waters but an additional, smaller peak in RPKM observed in mesopelagic depths, such as FRAM18_bin185 (NS5_B). Asides from depth, the geographical distribution patterns of species within and between genera varied (Supplementary Figs. S10–S14) which typically reflected distinct dynamics in relation to temperature (Supplementary Fig. S15), salinity (Supplementary Fig. S16) and oxygen (Supplementary Fig. S17). However, no clear patterns were evident with respect to nitrate + nitrite, phosphate, silicate or chlorophyll *a*. The geographical distribution patterns of species could be categorised into three types.

**Fig. 3:**
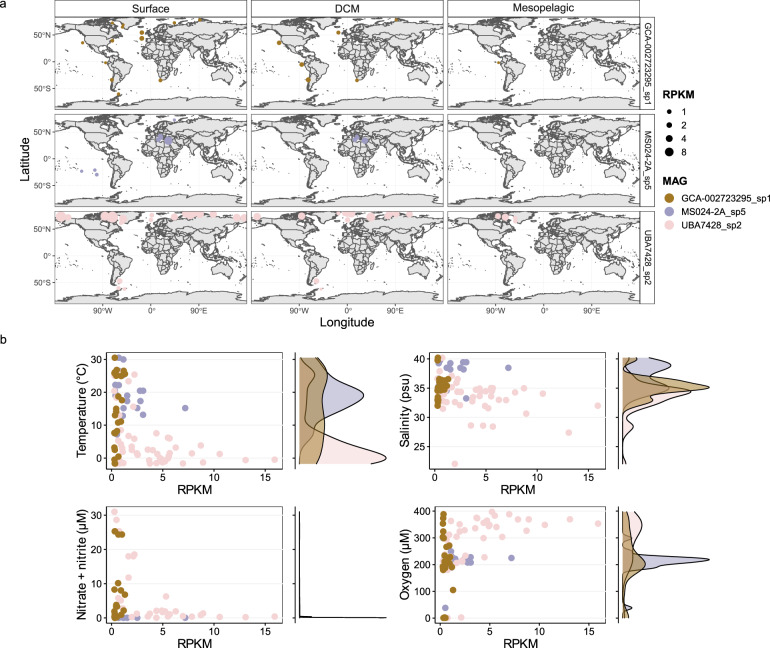
Three select species that represent different distribution types observed across the NS5 and their dynamics in relation to abiotic conditions. RPKM values were calculated based on read recruitment from Tara Oceans metagenomes to species-representative MAGs using BBMap with a 99% identity threshold. A minimum threshold of 0.25 RPKM was applied which ensured a minimum genome coverage of 40%. **a** Global distribution and **b** dynamics in species RPKM values across abiotic conditions. Within the scatter plots, each point represents a Tara Oceans sample where the species RPKM value was >0.25. Alongside each scatterplot is a density diagram showing the distribution of points.

The first, encompasses species-representatives with higher RPKM values in a specific geographical region, e.g. the Mediterranean and Red Sea for MS024-2A_sp5 (Fig. 3). As a result, representatives of this type exhibited narrow peaks in RPKM values in relation to abiotic conditions, e.g. for MS024-2A_sp5 at ~15 °C and ~38 psu. The second pattern is represented by changes in RPKM values with latitude, e.g. higher values in the Arctic for species UBA7428_sp2 (Fig. 3) and all species in NS5_F or in temperate regions for GCA-002723295_sp2 in NS5_B (Supplementary Fig. S11) and MS024-2A_sp7 in NS5_D (Supplementary Fig. S13). These species typically exhibited peak RPKM values within a defined range of each abiotic condition. For example, the Arctic-preference distribution of NS5_F species was related to peaks in RPKM values at temperatures <5 °C, oxygen concentrations >300 µM and salinities <33 psu whereas the temperate-preference distribution of UltraPac_E_bin_84_1 was related to peaks across a wide range of temperatures, 12–30 °C, and at salinity values of 33–38 psu. Lastly, the remaining species exhibited an unclear distribution pattern, either due to below-threshold RPKM values in most samples or comparable RPKM values in samples without a clear pattern, e.g. GCA-002723295_sp1 in NS5_B (Fig. 3). Representatives of this last distribution type, as could be expected, showed a lack of or an inconsistent pattern with shifts in abiotic conditions. Species RPKM values across all Tara Oceans samples are provided in Supplementary Table S4.

### Seasonal dynamics of NS5 species

**Fig. 4:**
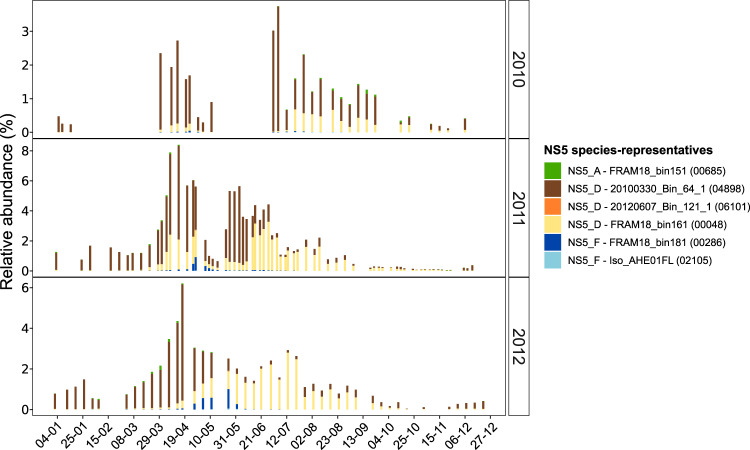
Temporal dynamics of NS5 species-representatives in surface waters at Helgoland Roads, North Sea. Distributions were obtained by recruiting oligotype representatives from a previously published dataset (62) to each species-representative MAG using BBMap with a 100% identity threshold. Next to each species-representative name, the original oligotype number is provided for direct comparison with previous dataset. Only recruitments successful with a 100% identity threshold are included. The relative abundances for each oligotype were taken from the original publication.

By performing read recruitment analysis of 16S rRNA gene oligotypes from a previously published time-series dataset, we were able to visualise the temporal dynamics of six NS5 species-representatives at Helgoland Roads, German Bight. Each of the identified oligotypes exhibited distinct and recurrent temporal dynamics over three consecutive years (Fig. 4), with three also showing a successional pattern from spring to summer. This succession began with 20100330_Bin_64_1 (NS5_D) that peaked from early to late spring (up to 4.5% of the community), followed by FRAM18_bin181 (NS5_F) in late spring and FRAM18_bin161 (NS5_D) that also peaked in late spring but persisted throughout summer (up to 3.5% of the community). Although the isolate, Iso_AHE01FL, was recovered from Helgoland Roads, the respective oligotype was present in low relative read abundance throughout the annual cycle (<0.1% of the community).

### Functional characterisation

In order to assess functional differences across the NS5 genera, the gene annotations that were consistent across the three most complete MAGs in each genus were compared (referred to as genus-level values from hereon). The necessary genes for glycolysis, gluconeogenesis, the pentose phosphate pathway, the tricarboxylic acid cycle and for the major components of the electron transport chain were identified in all genera, confirming an aerobic heterotrophic metabolism. An additional unifying feature was the presence of a green-light proteorhodopsin (PR), which is not found in low light conditions. Mechanisms for nitrogen and phosphorous metabolism were conserved across all groups and restricted to an ammonium transporter (Amt family) and nitrogen response regulatory proteins (e.g. NtrC) along with the ability to build and hydrolyse long chain polyphosphates with a polyphosphate kinase (*ppk*) and an exopolyphosphatase. In addition, all species contained a glycogen synthase gene, indicating the capacity to use glycogen as a storage molecule. In contrast, genes related to sulfur metabolism were not conserved across genera, with only the NS5_B harbouring the capacity for assimilatory sulfate reduction. The ability to synthesise riboflavin was conserved whilst all genera lacked the genes required for biotin, thiamine and vitamin B12 synthesis. In contrast, significant differences were evident with respect to substrate acquisition and degradation potential between NS5 genera.

**Fig. 5:**
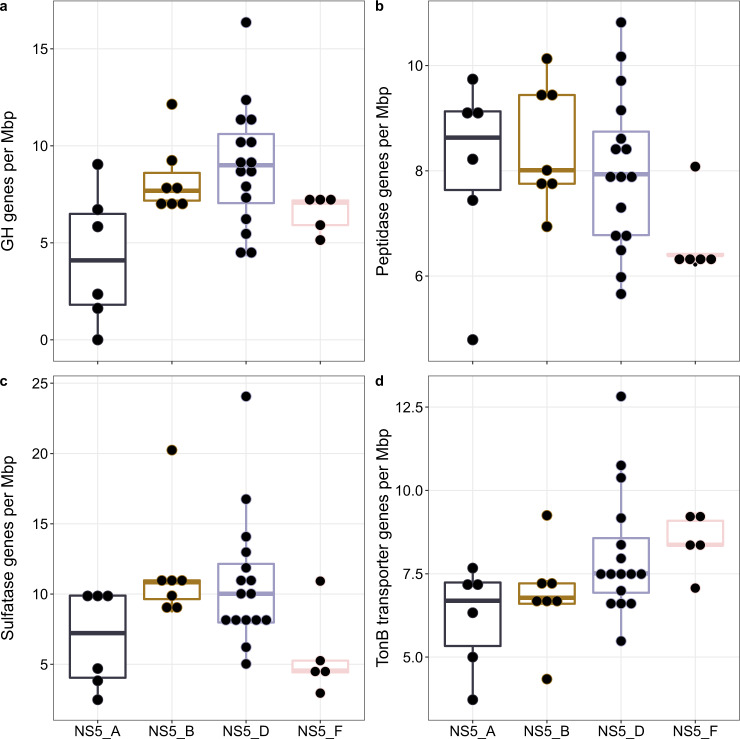
Summary of substrate utilisation genes annotated in species-representative MAGs. **a** Number of glycoside hydrolase genes based on agreeing annotations from HMMscan against dbCAN database and Diamond blastp search against the CAZy database. **b** Number of peptidases annotated using blastp search against the MEROPS database. **c** Number of sulfatase genes based on HMMscan against the Pfam sulfatase profile and blastp search against the SulfAtlas database. **d** Number of TonB-dependent transporters based on HMMscan against TIGRFAM TonB profiles.

The annotation of CAZymes varied considerably across genera and species (Supplementary Tables S5 and S6). The number of glycoside hydrolase (GH) genes across all species-representatives ranged from 0–12 per Mbp (Fig. 5 and Supplementary Table S5), whilst genus-level values ranged from 9 per Mbp in NS5_D and _B to 5 per Mbp in NS5_A. There were also clear distinctions in the composition of conserved and non-conserved GH gene families within each genus (Supplementary Fig. S18). The NS5_D harboured eight conserved gene family annotations compared to four in NS5_F, three in NS5_B and one in NS5_A. There were no universally conserved gene families. However, two conserved gene families were shared between the NS5_B, _D and _F, including a GH16_3 (β-1,3-glucanase) and a GH3. Conserved gene families specific to a single genus included GH29 and GH95 (both known as α-fucosidases) in NS5_D and GH113 (β-mannanase) in NS5_F. The large range in non-conserved GH gene family annotations across genera indicated a varying degree of substrate-metabolic diversity on the species-level (Supplementary Fig. S18). Most notable was the diversity within NS5_B, with 26 different GH gene families or sub-families. This also provided evidence that potential substrates, not conserved at the genus-level, are shared between species of different genera. For example, annotations for α-fucosidases were not restricted to NS5_D, but also found in some species of NS5_B (GH151) and NS5_F (GH107). The presence of GH16_3, GH18 and GH20 genes across species from all genera indicated a shared potential to degrade β-1,3-glucans, such as laminarin, and β-hexosamines, such as peptidoglycan. In addition, a number of annotations were unique to some species within a single genus, including GH43_1 (β-xylosidase/α-L-arabinofuranosidase) and GH142 (β-L-arabinofuranosidase) in NS5_B, GH13_31 (α-glucosidase), GH28 (α-L-arabinofuranosidase) and GH28 (poly-/rhamno-galacturonase) in NS5_D and GH144 (β-1,2-glucosidase) in NS5_F. Further comparisons on GH gene family annotations revealed that each species’ composition is unique (Supplementary Table S6).

A major process in carbohydrate catabolism in heterotrophic microbes involves glycan transport into the cell, a process mediated by, among others, TBDTs. The number of annotated TBDTs at the genus-level ranged from 7–9 per Mbp (Table 1) and at a species-level, from 4–13 per Mbp (Fig. 5 and Supplementary Table S5). In addition to TBDTs, the composition of all transporters was compared between genera (Supplementary Fig. S19). There were 23 universal transporters, including for vitamins and metals (Vitamin B12, zinc and magnesium), peptides (Di-tripeptide and D-serine) and carbohydrates (sodium/glucose, L-idonate, high-affinity gluconate and sugar SemiSWEET). In addition, 11 transporters were shared between more than one genus whilst 23 were unique to a single genus. The NS5_B and NS5_D both shared transporters indicative of more versatile metabolisms, including fatty acid and C4-dicarboxylate TRAP transporters.

**Table 1:** Genomic statistics and carbohydrate and peptide-degradation gene repertoire of NS5 genera.

	Genome completeness (%)	Genome contamination (%)	Genome size (Mbp)	GC content (%)	GHs/Mbp	CAZymes/Mbp	Peptidases/Mbp	Sulfatases/Mbp	TBDTs/Mbp	SusCs/Mbp	SusDs/Mbp	PULs	GH: peptidase	GH: sulfatase	GH: TBDT
NS5_A	81.4	0.3	1.17	30	5	7	8	8	7	2	3	0	1:1.6	1:1.6	1:1.4
NS5_B	97.3	0.5	1.82	30	9	12	8	13	8	2	2	2	1:0.9	1:1.4	1:0.9
NS5_D	99.1	1.4	2.02	37	9	12	7	9	9	4	4	3	1:0.8	1:1	1:1
NS5_F	97.0	0.1	2.05	36	7	10	7	4	9	2	3	2	1:1	1:0.6	1:1.3

Values are derived from the average of the three most complete metagenome-assembled genomes in each genus. Completeness and contamination were estimated using CheckM v1.1.2. Gene groups are shown as per Mbp values.

*TBDT* TonB-dependent transporters.

Distinct differences were also observed for sulfatase and peptidase gene annotations. The number of sulfatases ranged considerably, from 2 to 24 per Mbp across species (Fig. 5) and on the genus-level, from 4 per Mbp in NS5_F to 13 per Mbp in NS5_B (Table 1). In comparison, peptidases were more consistent at the genus-level, ranging from 7 to 8 per Mbp (Table 1) and exhibited a narrow range across species, 6–11 per Mbp (Fig. 5).

To provide an additional perspective on substrate preferences, the ratio of GH genes to other substrate utilisation genes was calculated (Table 1), a metric that has previously been employed for other flavobacterial groups [7]. The NS5_A consistently had the lowest ratios of GH genes and was the only genus to harbour less GH genes than peptidases, 1:1.6. The ratio of CAZymes:sulfatases also varied across genera, with NS5_F being the only genera to contain more GH genes than sulfatases (Table 1).

**Fig. 6:**
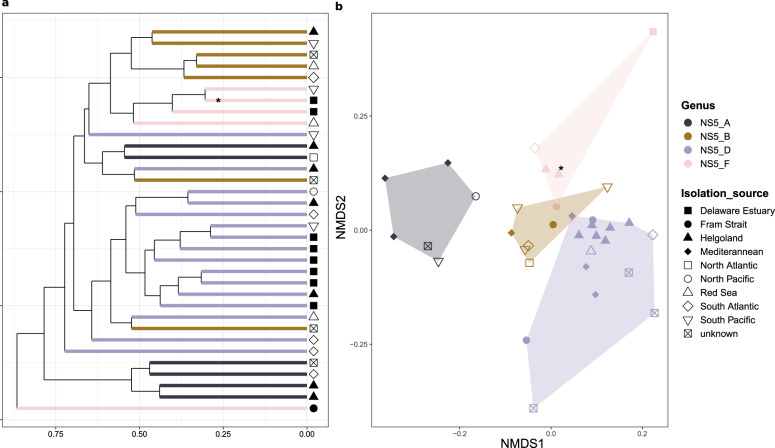
Comparison of the substrate utilisation gene composition between species-representatives. A dissimilarity matrix was generated from the CAZyme, peptidase, sulfatase and TonB-dependent transporter gene compositions and subsequently used for **a** hierarchical clustering analysis and **b** non-metric multi-dimensional scaling ordination. *indicates the isolate, Iso_AHE01FL.

Comparing the gene repertoire of CAZymes and peptidases across all species-representatives through a dissimilarity distance matrix approach, resulted in a clustering of species based on phylogeny (Fig. 6). This suggests that the substrate utilisation potential is primarily determined through evolution, and not an adaptation to habitats based on lateral gene transfer. However, refining the dataset to only contain specific sets of genes, e.g. only CAZymes, resulted in less coherent phylogeny-based clustering, although the effects across genera varied depending upon the chosen gene set (Supplementary Fig. S20).

### Polysaccharide utilisation loci (PULs) and SusC/SusD protein trees

PULs are genetic clusters of functionally related genes that are involved in the binding and cleavage of polysaccharides and subsequent uptake of oligosaccharides into the cell [80]. Canonical PULs are those containing degradative CAZymes and a SusC/SusD gene pair [80], which provides the transport mechanism for large oligosaccharides across the outer membrane. However, atypical or non-canonical PULs, lacking the SusC/SusD gene pair but still consisting of numerous degradative CAZymes, have also been described [81]. PULs are typically specific towards certain polysaccharides and can thus provide valuable information on variations in substrate metabolism across species, even if the SusC/SusD gene pair is absent. The total number of PULs across NS5 species ranged from 0 to 6, with an almost complete absence in NS5_A (Table 1 and Supplementary Table S7).

**Fig. 7:**
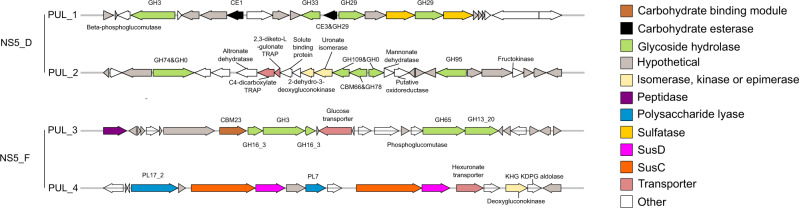
Polysaccharide utilisation loci containing gene colocalisations that are conserved within candidate genera. The structures presented include two each from the candidate species of NS5_D, 20100330_Bin_64_1, and NS5_F, Iso_AHE01FL. Although the exact PUL structures vary across species, the colocalisation of CAZymes are conserved within all species of the genus. The predicted substrate targets for NS5_D PULs are, PUL_1 = α-fucan and PUL_2 = unclear, and for NS5_F, PUL_3 = laminarin and PUL_4 = alginate.

PUL structures were highly diverse across species, but four examples of conserved gene colocalisations were identified at the genus-level, two in NS5_D and two in NS5_F (Fig. 7). In NS5_D, one conserved colocalisation consisted of several GH29 genes (α-fucosidases), a single GH33 (sialidase) and/or GH3 gene and at least two sulfatases. Additionally, species within NS5_D harboured a PUL containing a solute-binding protein, C4-dicarboxylate TRAP transporter, 2,3-diketo-L-gulonate TRAP transporter, uronate isomerase and mannonate dehydratase which was accompanied by a GH95 and fructokinase gene in many of the representatives. Such a structure was also identified in one MAG from NS5_B, UltraPac_3515_G_bin_18, and indicates an ability to uptake sugar acids and C4 carbon compounds. In the NS5_F, one conserved colocalisation consisted of several GH16_3 (β-1,3-glucosidase) genes with a GH3 gene, with all but one MAG also encoding for a GH109 (N-acetylhexosaminidase) and galactokinase gene in the same region. In Iso_AHE01FL, this was further supplemented by a sodium/glucose cotransporter, a GH65 and β-phosphoglucomutase gene, providing additional machinery for β-glucan degradation. The second conserved colocalisation in NS5_F consisted of a double SusC/SusD gene pair along with at least two polysaccharide lyase (PL) genes from the PL6, PL7 and PL17 families, suggesting alginate as a potential substrate target (Fig. 7).

The reconstruction of protein trees using SusC and SusD genes additionally confirmed the predicted substrate targets of PULs (Supplementary Fig. S21). For example, the SusC and SusD genes derived from potential alginate-targeting PULs identified in NS5_F species, clustered with those from alginate-targeting PULs of previously recovered MAGs and cultured isolates of *Flavobacteriia*.

### Novel candidate genera

Based on the phylogenetic and ecological partitioning of NS5 species, sufficient information has been collected to formally describe four candidate species and genera within the *Flavobacteriaceae* family, *Candidatus* Marisimplicoccus (NS5_A), *Candidatus* Marivariicella (NS5_B), *Candidatus* Maricapacicella (NS5_D) and *Candidatus* Arcticimaribacter (NS5_F). The etymology and metabolic descriptions of these are provided in Supplementary Material S1 and Supplementary Figs. S22–S25.

## Discussion

The NS5 marine group represent one of the most prevalent groups of marine flavobacteria across the world’s oceans yet our knowledge on their phylogenetic diversity and ecological functions is limited. Here, we phylogenetically and ecologically characterise four novel candidate genera within the NS5, each with a candidate representative species. The genera encapsulate 35 distinct species that are distinguishable by genomic characteristics, spatiotemporal distribution patterns and predicted functional potentials, from which we can hypothesise ecological niche partitioning. Furthermore, we present the first complete genome sequence and morphological description of an NS5 isolate, “*Arcticimaribacter forsetii* AHE01FL”.

### Phylogeny and genomic characteristics

Phylogenetic tree reconstructions revealed four distinct, coherent genera in the NS5, each with multiple species-representatives, which formed a novel branch within the *Flavobacteriaceae* family. It is clear from tree topologies that two additional genera likely also exist, but a lack of representative genomes hinders further investigation.

NS5 species-representatives across genera are distinguishable by their genomic characteristics. The larger, average genome size of NS5_F and NS5_D (2.05 ± 0.19 and 2.02 ± 0.38 Mbp, respectively) are above the median size reported for a dataset of >1200 marine *Bacteroidetes* MAGs with comparable completeness values (1.96 Mbp) [38] whilst NS5_A is within the smallest 10% of genome sizes from that dataset. In general, the genome size of NS5 representatives are smaller than those of related cultured marine *Flavobacteriia*, such as *Polaribacter* (3.1–4.0 Mbp), *Tenacibaculum* (3.2–5.5 Mbp) and *Formosa* sp. *B* (2.7 Mbp). Genome sizes of cultured isolates and MAGs has previously been shown to vary by up to 1 Mbp in *Polaribacter* [7], however, the genome sizes of NS5_F MAGs were comparable to “*Arcticimaribacter forsetii* AHE01FL” (2.03 Mbp) in the same genus. Furthermore, within NS5_D, one of the species-representatives, MS024-2A_sp8, is a high quality draft single cell genome [16] which also has a comparable genome size to MAGs within the same genus. Therefore, the smaller genome sizes are likely not a methodological artefact but reflect differences in the life strategy and ecological role of NS5 compared to the other well described *Flavobacteriia*.

### Life strategy and metabolism

The major metabolic pathways and cellular functions were largely conserved across all four newly described candidate genera and indicative of an aerobic photoheterotrophic lifestyle with supplemental energy acquisition through a proteorhodopsin (PR). PRs are light-driven proton pumps that can generate ATP through the proton motive force [82]. PR-mediated photoheterotrophy is widely distributed among marine Archaea and Bacteria inhabiting the photic zone [83], and it has been shown that PR-containing marine flavobacteria have smaller genomes than PR-lacking flavobacteria [84]. Such findings are in-line with the genome sizes reported here. Another key finding of this study is that NS5 species exhibit free-living lifestyles, evidenced by a lack of flagella machineries and gliding motility and a distinct separation of visualised cells from particles. This is in agreement with previous studies that reported an enrichment of NS5 marine group in the free-living fraction (<3 µm) in the North Sea [85] and Southern Ocean [18].

For free-living aerobic heterotrophs, the main source of carbon and nutrients for growth is dissolved organic matter (DOM). As is known for other groups of marine *Flavobacteriia* [84, 86], NS5 species are shown to encode a suite of degradative CAZymes, indicating a specialised capacity for high molecular-weight DOM degradation. The number of GH genes in NS5_B, _D and _F representatives, 7–9 per Mbp, is similar to values reported for MAGs classified in the *Polaribacter* 1-b (9 per Mbp) and 2-a clusters (9 per Mbp) as well as some cultured isolates such as Formosa B (7.7 per Mbp) [86] and *Gramella forsetii* KT0803^T^ (10.5 per Mbp) [87]. These organisms are known as specialist degraders of algal-derived carbohydrates and are key members of microbial communities following spring phytoplankton blooms [86, 88]. In contrast, the number of peptidases present in NS5 species is considerably lower, 7–8 per Mbp, than in *Gramella forsetii* KT0803^T^, 30.5 per Mbp, and *Formosa B*, 25 per Mbp, indicating a reduced capacity for protein hydrolysis. Furthermore, the number of canonical PULs in NS5 species is lower than the average recently reported for a large dataset of marine *Bacteroidetes* MAGs [38]. Such features may be evidence of narrow substrate niches for NS5 species, which has also been suggested previously [16].

### Unique substrate-degradation potentials

NS5 species harbour distinct substrate utilisation capacities, with evidence of genus-wide conserved substrate targets also evident in NS5_D and _F. In NS5_F, these consist of laminarin and alginate. Laminarin is a major storage polysaccharide in marine diatoms and a common substrate of marine flavobacteria, based on the widespread presence of PULs [38] and rapid hydrolysis rates in incubations [89]. The NS5_F laminarin-targeting PULs resemble those previously described for *Gramella forsetii* KT0803^T^ [75], *Gramella* sp. MAR_2010_147 and *Gillisia* spp. Hel1_29 and Hel1_33_132 [75], shown to be upregulated in the presence of laminarin [88]. Alginate, also a widely available polysaccharide, constitutes a key component in brown algal cell walls, representing up to 45% of their dry weight biomass. Alginate-targeting PULs have been identified in a number of marine *Flavobacteriia* [75], however NS5 representatives dominated the alginate PUL cluster in the Helgoland Roads time-series dataset [38]. These PULs contain the Aly (PL6 and PL7) and Oal families (PL15 and PL17) that together, provide the capacity for complete alginate degradation [90, 91]. Additional substrate targets, shared by a minority of, or unique to a single species, also included algal-derived polysaccharides, such as α-fucan (GH107) for “*Arcticimaribacter forsetii* AHE01FL”.

A conserved PUL structure identified in species of NS5_D, containing α-fucosidases and sulfatases, suggests a potential to utilise fucose-containing sulphated polysaccharides (FCSP). However, previous FCSP-targeting PULs identified in fourteen isolates of marine *Flavobacteriia*, encoded a more extensive CAZyme gene repertoire, reflecting the complexity of FCSP [92, 93]. It is thus unlikely that NS5_D species utilise FCSPs but instead, cleave fucose groups bound to other carbohydrate structures or hydrolyse less complex fucose-containing oligosaccharides in a scavenging-like mechanism. Additionally conserved within NS5_D species, is a genetic loci containing numerous transporters for amino acids, D-xylose, acid sugars and C4 compounds without any degradative CAZymes. Such a structure may be evidence of genome rearrangement to increase the efficiency of gene regulation for substrate acquisition under certain conditions.

A lack of intra-genus conserved gene colocalisations were found in the NS5_B, however, all species contained a PUL targeting bacterial-derived polysaccharides, such as glycogen. In addition, unique PUL structures targeting algal-derived polysaccharides were identified in some species, such as an α-mannose-targeting PUL in GCA-002723295_sp2 (GH95). In NS5_A species, PULs were either absent or low in number and the comparable number of CAZyme to peptidase genes, which was unique to this genus, suggests that proteins are a more important substrate for growth.

### Ecological niche partitioning of species

Niche concept has been applied in marine microbial ecology to describe the partitioning of taxa either based on adaptations to specific conditions across environments [4, 6, 94] or adaptations to specialised substrates within an environment [5, 14]. Using time-series data from a coastal ecosystem, it has been shown that populations of *Bacteroidetes* occupy distinct substrate specific niches that drive recurrent temporal dynamics [7, 13, 14]. For the NS5 representatives identified in that dataset, several specific substrate targets were reported, including β-glucan, α-glucan, α-mannan and alginate [38]. We show in this study that these substrates are indicative of different genera. Furthermore, by using an oligotype dataset from the same time-series, we identified successional-like dynamics for some NS5 species. Those dynamics were also likely driven by substrate utilisation capacity, with the early spring responder, 20100330_Bin_64_1 (NS5_D), encoding for twice as many GH genes and sulfatases as well as a broader diversity of predicted substrate targets than the late spring responder, FRAM18_bin161 (NS5_D). This suggests that substrate may be a major factor in the niche partitioning of these species in that environment. It is important to note however, that other factors, not assessed in this study, likely also contribute to these temporal dynamics, such as grazing by microeukaryotes and viral-induced mortality.

Although substrate may act as a key niche-determining factor for NS5 species in a given environment, we show that species’ spatial distribution dynamics across environments and throughout the water column are influenced by distinct shifts in abiotic conditions. Depth, and the associated changes in light and temperature, is well evidenced to structure the vertical distribution of microbial taxa [8]. Such a pattern is also clear for NS5, with nearly all species showing a preference for the upper euphotic zone (<100 m). Adaptations to this environment are evident within the genomes and predicted metabolisms of NS5 species, e.g. the presence of PR and utilisation of HMW-DOM as a substrate that is primarily produced by, or a result of lysis of phytoplankton in the euphotic zone. On geographical spatial scales, studies on microbial biogeography have reported that temperature and oxygen are the strongest correlates to changes in taxonomic and functional composition [95, 96]. The distribution dynamics of NS5 species are in agreement with this, although distinct patterns could also be identified in relation to salinity. It is clear that the niche-determining conditions vary considerably across species, with adaptations to narrow and broad ranges of conditions observed.

The widespread presence of many NS5 species but with distinct preferences for specific environmental conditions are in support of previous theories such as “everything is everywhere but the environment selects” [97] and the microbial seed bank hypothesis [98, 99]. The capacity to survive “everywhere” likely reflects an evolutionary adaptation that resulted in small genomes and cell sizes with advantageous features such as a PR and a potential to utilise widely available substrates. However, it is clear that each species has adapted to a specific set of conditions under which it can proliferate within its defined ecological niche. The factors that determine the partitioning of niches for NS5 species is a combination of abiotic conditions, such as temperature, and substrate utilisation. We propose that abiotic conditions influence spatial and temporal niche space across environments for each species, whereas substrate availability most strongly influences temporal niche dynamics within an environment.

We recognise that additional factors, particularly biotic interactions, can play an important role in determining a species’ niche, but we were unable to address these in the scope of this study. Thus, further work would be required to understand the influence these factors have.

We present evidence here that NS5 genera are distinguishable by phylogeny, cell shape and size, genomic characteristics, spatiotemporal distribution patterns and predicted substrate metabolism. Based on this, we formally describe four novel candidate genera and type species within the *Flavobacteriaceae* family—etymology and metabolic descriptions are provided in Supplementary Material S1 and Supplementary Figs. S22–S25.

## Supplementary information

Supplementary material S1

Supplementary figures all

Supplementary tables all

## Data Availability

Metagenomes and MAGs used in this study were either previously deposited or deposited for this study in the European Nucleotide Archive, with all accession numbers provided in Supplementary Table S2. Those deposited for this study include the cultured isolate genome, PRJEB45371, and the MAGs derived from the South Pacific gyre metagenomes, PRJEB43746. The 16S rRNA gene amplicon time-series dataset used was previously published [63] and stored by JGI in the GOLD database under the project ID Gp0056779 as part of the community sequencing project COGITO. The ARB database containing the 16S rRNA gene NS5 phylogenetic tree is provided as Supplementary File S1. All data tables and the R script required to recreate the main body figures are available at https://github.com/tpriest0/NS5_marine_group_manuscript_figures.
