# Supplementary material for: Niche partitioning of the ubiquitous and ecologically relevant NS5 marine group
Source: ISME J. 2022 Feb 15;16(6):1570–82. doi: 10.1038/s41396-022-01209-8 (PMC9122927; doi:10.1038/s41396-022-01209-8)
Supplement: Supplementary file 1 — Supplementary material S1 [file 41396_2022_1209_MOESM1_ESM.pdf]

Supplementary Material 1

Supplementary Methods

Fram Strait sampling and sequencing

Seawater samples were collected at the deep chlorophyll maximum layer from eleven stations across the Fram Strait region in July and August 2018 during the PS114 Polarstern cruise, as described previously [1]. In short, seawater was retrieved using a CTD Rosette sampler and 500 ml was sequentially filtered through 10, 3 and 0.2 µm pore-size polycarbonate membrane filters. The genomic material was extracted from the 0.2 – 3 µm fraction using a modified SDS-based extraction method after Zhou *et al* [2] and the DNA subsequently sequenced at the Max Planck Genome Centre in Cologne, Germany. The sequencing was performed on an Illumina HiSeq 3000 (2 x 150 bp) and PacBio Sequel II (Hifi reads) platform. Quality trimming and processing of raw reads was performed as previously described [1].

Generation of MAG dataset

The metagenomic reads from the Fram Strait samples were assembled, binned and manually refined as described previously [1]. Briefly, Illumina reads were assembled using Megahit v1.2.9 (parameters: --presets meta-large, --cleaning-rounds 5) [3] whilst metaFlye v2.6.0 [4] was used to assemble PacBio reads. For both datasets, contigs were binned using Concoct [5], Metabat2 [6] and Maxbin2 [7] with default settings and a consensus set of bins generated using DASTool [8]. To improve the contiguity and size of the generated bins, taxonomic reassembling was performed on a class level. All MAGs were then manually refined using Anvio v6.1 [9] and clustered into species based on a 95% average nucleotide identity threshold with FastANI v1.9 [10]. Species-representative MAGs were classified, where possible, using 16S rRNA gene phylogeny, as described previously [1]. In short, 16S rRNA gene sequences were extracted from species-representative MAGs using Barnnap v0.9 [11] and placed into the SILVA 138 SSU NR99 reference phylogenetic tree [12, 13] using the SINA aligner [14] and Maximum Parsimony algorithm of the ARB program [15].

NS5 MAG phylogenetic tree reconstruction

The 16 ribosomal proteins used for phylogenetic tree reconstruction included L2, L3, L4, L5, L6, L14, L16, L18, L22, L24, S3, S8, S10, S17, S19 [16]. To serve as an outgroup, 15 species-representative genomes of *Sphingobacteriia* were downloaded from the RefSeq database. Coding sequences were predicted with Prodigal v2.6.3 [17] and the ribosomal target proteins were identified with HMMsearch v3.3.1 (hmmer.org) against Pfam HMM models for each protein

(E-value 1 x 10^{-5}). The gene sets were aligned individually using Muscle v3.8.15 (parameters: -maxiters 24) [18] and conserved gaps and ambiguously aligned regions were trimmed with TrimAI v1.4.1 (parameters: -automated1) [19]. The 16 individual gene alignments were concatenated to form a single alignment using the concat script from BinSanity [20]. FastTree v2.1.10 (parameters: -gamma –lg) [21] was used to construct a phylogenetic tree that was visualised and annotated using the Interactive Tree of Life v4 (IToL) [22].

To determine the stability of the identified NS5 phylogeny and visualise the position of this group within the larger *Flavobacteriia*, an additional 102 genomes and 1273 assemblies labelled as 'Complete' and 'Representative' were retrieved from the RefSeq database. A phylogenetic tree was reconstructed following the same workflow as described above, with 20 species-representative genomes assigned to the *Alphaproteobacteria* class as an outgroup.

Probe design and environmental cell visualisation

Oligonucleotide probes were designed in ARB using the complete SILVA 138 SSU NR99 database, along with the MAG-derived sequences, as a reference. The in-built 'probe design' tool within ARB failed to identify possible probe target regions and as such, probes were manually designed by visually inspecting the alignment. Due to high sequence similarity to neighbouring groups within the *Flavobacteriaceae*, probes were successfully designed for only NS5_A and NS5_F. The probes were subsequently synthesised by biomers.net with a horseradish peroxidase enzyme ligated to the 5' end, generating catalysed reporter deposition-fluorescence *in situ* hybridization probes. Optimal hybridisation conditions for each probe were determined by testing with varying formamide concentrations (0 – 60%) at a temperature of 46 °C on filtered water samples from the Fram Strait dataset [1]. The chosen formamide concentration for each probe was determined as the highest concentration that provided sufficient brightness for signal detection. The probes were subsequently applied to five filtered surface seawater samples (0.2 – 3 µm fraction) from the Fram Strait region derived from [1]; sample names used in the figure are retained from that study for ease of comparison.

Seasonal dynamics of species-representative MAGs

A multiyear sampling campaign of surface waters at Helgoland Roads, North Sea, resulted in a comprehensive 16S rRNA gene tag dataset that has been previously published and described in detail [23]. In short, surface water samples were collected at a depth of ~1 m and size fractionated using filtration into a 0.2 – 3 µm and 3 – 10 µm fraction. For our analysis, we only used the data derived from the 0.2 – 3 µm fraction. Amplicons of the samples were generated by amplification of the V4 region of the 16S rRNA gene with the primers 515 F (5′-

GTGCCAGCMGCCGCGGTAA-3′) and 806 R (5′-GGACTACHVGGGTWTCTAAT-3′). Amplicons were sequenced on an Illumina MiSeq platform with 2 x 250 bp chemistry at the Department of Energy Joint Genome Institute (DOE-JGI, Walnut Creek, CA, USA). The raw sequence data from the 0.2 – 3 μm fraction is stored in the GOLD database under the project ID 'Gp0056779'. Amplicons were processed using minimum entropy decomposition (MED) [24], as described previously [23]. The relative abundance of individual oligotypes was then estimated as the proportion of reads from each sample matching the representative oligotype sequence, after removal of sequences classified as chloroplast, mitochondria or no relative. The resulting MED node representatives, or oligotypes, were recruited to NS5 species-representative MAGs using BBMap with a 100% threshold (minid=100, idfilter=100). The relative abundance dynamics of successfully mapped oligotypes was then visualised using Rstudio with the vegan and ggplot2 packages.

Comparative analysis of RPKM values

As read recruitment and calculation of metrics like RPKM can be subject to bias, we compared RPKM values to cell counts (see Probe design and environmental visualisation section above) and another metric, the truncated average sequence depth (TAD, see [25]), to provide additional support. TAD values were calculated for selected MAGs that showed large RPKM value ranges, by re-recruiting reads from Tara Oceans metagenomes. For each MAG, nine Tara Oceans metagenomes were selected which represented high, medium and low RPKM values for the respective MAG. Scatter plots were produced for RPKM vs TAD values and RPKM vs coverage, whilst barcharts were used to visualise cell counts vs RPKM values; all produced using ggplot2.

Results

Comparative analysis of RPKM values

Calculation of RPKM values relies on read recruitment which is subject to bias, e.g. high coverage of conserved regions. To provide support for RPKM values in this study, we compared RPKM values to environmental cell counts in the same samples as well as to another, more robust metric, the truncated average depth. As a result of these comparisons, RPKM values below 0.25 were removed from analyses, as these values typically related to low coverage of genomes from read recruitment (Supplementary Figure S6). Above this threshold, RPKM values related to coverage across the genomes of at least 40% and linearly related to TAD values

(Supplementary Figure S17). Furthermore, fluctuations in RPKM values reflected those in cell counts obtained from CARD-FISH (Supplementary Figure S4).

Candidate genus and species information

NS5_A – *Candidatus* Marisimplicoccus

Ma.ri.sim.pli.coc'cus. L. neut. n. *mare*, the sea; L. masc. adj. *simplus*, simple; N.L. masc. n. *coccus* (from Gr. masc. n. *kokkos*), a grain or a berry; N.L. masc. n. *Marisimplicoccus*, a simple coccus from the sea.

Candidatus Marisimplicoccus contains marine aerobic heterotrophic bacteria that have small genomes (average of 1.17 Mbp) and a low GC content (~30%). With the currently available sequence data, six species are discernible. Through the application of a newly designed oligonucleotide CARD-FISH probe (Supplementary Table S3), cells within this genus are identified as coccoid in shape (Figure 2), with a small diameter, <0.5 µm. *Candidatus* Marisimplicoccus are distributed across all oceanic regions analysed, with each species exhibiting different distribution patterns. Most common across the distribution patterns is an increased presence in the North Atlantic region and Chilean upwelling system. Apart from MED-G11_sp2, all species decrease in prevalence from the photic to mesopelagic zone.

The substrate-based metabolic gene repertoire consists of a low number of degradative CAZymes and a high peptidase to GH gene proportion. The only predicted genus-wide metabolisable carbohydrates are N-acetylglucosamines (GH109). The former identification of this genus is MED-G11. The type species is *Candidatus* Marisimplicoccus framensis and the corresponding type material is the metagenome-assembled genome FRAM18_bin151.

Description of *Candidatus* Marisimplicoccus framensis

fra.men'sis; N.L. masc. adj. *framensis*, of Fram Strait, corresponding to the origin of the type species material from the Fram Strait region.

The type material of this species is a metagenome-assembled genome recovered from a sample taken at the deep chlorophyll maximum in the Fram Strait region in 2018 (Biosample accession: SAMEA7768515, Genbank assembly accession: GCA_905182065.1). The genome size is 1.232 Mbp with a GC content of 30% and the genome assembly contains a complete 16S rRNA (1525 bp), 23S rRNA (2813 bp) and 5S rRNA (103 bp) gene and 31 tRNA genes. A schematic overview of the metabolism of this species is provided in Supplementary Figure S22.

NS5_B - *Candidatus* Marivariicella

Ma.ri.va.ri.i.cel'la. L. neut. n. *mare*, the sea; L. masc. adj. *varius,* varying or versatile; L. fem. n. *cella*, a cell; N.L. fem. n. *Marivariicella*, the varying cell of the sea

Organisms within *Marivariicella* are marine aerobic heterotrophs with an average genome size of 1.821 Mbp and GC content of 30%. There are currently seven discernible species that exhibit distinct distribution patterns across the world's oceans but each with a decrease in prevalence from the surface to mesopelagic zone. All species within this group contain a proteorhodopsin and the genes required for sulfate assimilation, a unique feature to this genus. The identifiable substrate targets conserved across at least 90% of species in this genus include α- / β- N-acetylhexosamine (GH109 and GH20), β-1,3-glucan (GH16_3) and peptidoglycan (GH18 and/or GH73 and/or GH23). Additionally, the high number of sulfatase to GH genes indicates an adaptation to metabolise sulfated polysaccharides. Despite having only a small set of conserved substrates, the variation in metabolic capabilities across species is high. The previous identification of this genus is GCA-002723295. The type species is *Candidatus* Marivariicella framensis and the corresponding type material is the metagenome-assembled genome FRAM18_bin185.

Description of *Candidatus* Marivariicella framensis

fra.men'sis; N.L. fem. adj. *framensis*, of Fram Strait, corresponding to the origin of the type species material from the Fram Strait region.

The type material, FRAM18_bin185, is a metagenome-assembled genome derived from a water sample taken in the Fram Strait region in 2018 (Biosample accession: SAMEA7768595, Genbank assembly accession: GCA_905182735.1). The assembly is of high quality with a completeness of 99.26% and 0% contamination and contains a partial 16S rRNA (986 bp), 23S rRNA (829 bp) and a 5S rRNA (105 bp) gene along with 31 tRNA genes. The genome size is 1.914 Mbp with a GC content of 30.7%. A schematic overview of the metabolism of *Candidatus* Marivariicella framensis is provided in Supplementary Figure S23.

***NS5_D* – *Candidatus* Maricapacicella**

Ma.ri.ca.pa.ci.cel'la; L. neut. n. *mare* (*gen. maris*), the sea; L. masc. adj. *capax,* capable; L. fem. n. *cella*, a cell; N.L. fem. n. *Maricapacicella*, a capable cell of the sea

Within *Candidatus* Maricapacicella, there are currently 16 discernible species of marine aerobic heterotrophs with an average genome size of 2.03 Mbp and GC content of 37%. Global distribution patterns on a genus-level indicate a preference for Arctic over temperate or tropical

regions and a higher prevalence in the photic zone compared to the mesopelagic zone however, the distribution patterns of species is highly variable and indicative of habitat preferences. Members of this genus appear to be metabolically versatile, capable of degrading several different high-molecular weight carbohydrates as well as uptake acid sugars and C4 compounds. The identifiable conserved substrate targets across the genus include α- / β- N-acetylhexosamine (GH109 and GH20) and α-L-fucose containing compounds (GH29 and GH95). Additionally, the ability to degrade β-1,3-glucan (GH16_3) and α-glucans (GH65) is widely present along with a broad range of additional substrates specific to individual species. The previous identification of this genus is MS024-2A. The type species is *Candidatus* Maricapacicella forsetii and the corresponding type material is the metagenome assembled genome 20120607_Bin_121_1

Description of *Candidatus* Maricapacicella forsetii

for.se'ti.i; N.L. gen. masc. n. *forsetii,* of Forseti, Scandinavian god of justice and reconciliation resident on Helgoland, from where the genome was recovered.

The type material, 20100330_Bin_64_1, is a metagenome-assembled genome derived from a water sample taken at Helgoland, Germany, during a spring phytoplankton bloom in 2012 (Biosample accession: SAMEA5403668). The assembly is of high quality with a completeness of 100% and 1.84% contamination and contains a complete 16S rRNA, a partial 23S rRNA (1353 bp) and two 5S rRNA genes along with 36 tRNA genes. The genome size is 2.003 Mbp with a GC content of 35.7%. A schematic overview of the metabolism of *Candidatus* Maricapacicella forsetii is provided in Supplementary Figure S24.

***NS5_F – Candidatus* Arcticimaribacter**

Arc.ti.ci.ma.ri.bac'ter. L. masc. adj. *arcticus*, northern, arctic; L. neut. n. *mare*, the sea; N.L. masc. n. *bacter*, rod or staff; N.L. masc. n. *Arcticimaribacter,* an Arctic sea rod

The *Candidatus* Arcticimaribacter genus is a group of marine aerobic heterotrophic bacteria that contains five species with an average genome size of 2.03 Mbp and a GC content of 36%. All species are globally present from the surface to mesopelagic zones but two distinct distribution patterns are evident within the genus: three species show a preference for Arctic environments whilst the other two exhibit a cosmopolitan-like distribution. Annotation of genes involved in substrate-metabolism reveals an equal number of GH genes and peptidases but with a low number of sulfatases, suggesting a preference for unsulfated polysaccharides and proteins as a substrate. The conserved identifiable substrate targets across the genus are β-1,3-glucan

(GH16_3), α- / β- N-acetylhexosamine (GH109 and GH20) and alginate (PL6, PL7, PL17), with additional, unique substrate targets present across species. Cells within this genus are rod-shaped with a length of 0.5 – 1 µm, visualised using CARD-FISH on environmental samples and electron microscopy of a cultured isolate. The previous identification of this genus is UBA7428. For this genus, the type species is *Candidatus* Arcticimaribacter forsetii which is represented by a complete genome sequence from a cultured isolate (strain name *Flavobacteriales* bacterium AHE01FL).

Description of *Candidatus* Arcticimaribacter forsetii

for.se'ti.i; N.L. gen. masc. n. *forsetii,* of Forseti, Scandinavian god of justice and reconciliation resident on Helgoland, from where the genome was recovered.

The type material, *Flavobacteriales* bacterium AHE01FL, is a complete genome sequence from a pure cultured isolate recovered from Helgoland Roads, Germany, in 2016 (Biosample accession: SAMEA5403668). The assembly is of closed circular genome with a CheckM completeness of 94.7% and 0.18% contamination and contains two complete 16S rRNA, 23S rRNA (1353 bp) and 5S rRNA genes along with 37 tRNA genes. The genome size is 2.034 Mbp with a GC content of 34.15%. This organism is a rod shaped bacterium with a length of up to 1 µm and width <0.5µm (Figure 2). A schematic overview of the metabolism of *Candidatus* Arcticimaribacter forsetii is provided in Supplementary Figure S25.

References

1. Priest T, Orellana LH, Huettel B, Fuchs BM, Amann R. Microbial metagenome-assembled genomes of the Fram Strait from short and long read sequencing platforms. *PeerJ* 2021; **9**: e11721.
2. Zhou J, Bruns MA, Tiedje JM. DNA recovery from soils of diverse composition. *Applied and environmental microbiology* 1996; **62**: 316–322.
3. Li D, Luo R, Liu C-M, Leung C-M, Ting H-F, Sadakane K, et al. MEGAHIT v1.0: A fast and scalable metagenome assembler driven by advanced methodologies and community practices. *Methods* 2016; **102**: 3–11.

4. Kolmogorov M, Bickhart DM, Behsaz B, Gurevich A, Rayko M, Shin SB, et al. metaFlye: scalable long-read metagenome assembly using repeat graphs. *Nature Methods* 2020; **17**: 1103–1110.
5. Alneberg J, Bjarnason BS, de Bruijn I, Schirmer M, Quick J, Ijaz UZ, et al. Binning metagenomic contigs by coverage and composition. *Nature Methods* 2014; **11**: 1144–1146.
6. Kang DD, Li F, Kirton E, Thomas A, Egan R, An H, et al. MetaBAT 2: an adaptive binning algorithm for robust and efficient genome reconstruction from metagenome assemblies. *PeerJ* 2019; **7**: e7359.
7. Wu Y-W, Simmons BA, Singer SW. MaxBin 2.0: an automated binning algorithm to recover genomes from multiple metagenomic datasets. *Bioinformatics* 2016; **32**: 605–607.
8. Sieber CMK, Probst AJ, Sharrar A, Thomas BC, Hess M, Tringe SG, et al. Recovery of genomes from metagenomes via a dereplication, aggregation and scoring strategy. *Nature Microbiology* 2018; **3**: 836–843.
9. Eren AM, Esen ÖC, Quince C, Vineis JH, Morrison HG, Sogin ML, et al. Anvi'o: an advanced analysis and visualization platform for 'omics data. *PeerJ* 2015; **3**: e1319.
10. Jain C, Rodriguez-R LM, Phillippy AM, Konstantinidis KT, Aluru S. High throughput ANI analysis of 90K prokaryotic genomes reveals clear species boundaries. *Nature Communications* 2018; **9**: 5114.
11. Seeman T. barrnap 0.9: rapid ribosomal RNA prediction.
12. Quast C, Pruesse E, Yilmaz P, Gerken J, Schweer T, Yarza P, et al. The SILVA ribosomal RNA gene database project: improved data processing and web-based tools. *Nucleic Acids Res* 2013; **41**: D590–D596.
13. Yilmaz P, Parfrey LW, Yarza P, Gerken J, Pruesse E, Quast C, et al. The SILVA and "All-species Living Tree Project (LTP)" taxonomic frameworks. *Nucleic Acids Res* 2014; **42**: D643–D648.

14. Pruesse E, Peplies J, Glöckner FO. SINA: accurate high-throughput multiple sequence alignment of ribosomal RNA genes. *Bioinformatics* 2012; **28**: 1823–1829.
15. Ludwig W, Strunk O, Westram R, Richter L, Meier H, Yadhukumar, et al. ARB: a software environment for sequence data. *Nucleic Acids Res* 2004; **32**: 1363–1371.
16. Hug LA, Baker BJ, Anantharaman K, Brown CT, Probst AJ, Castelle CJ, et al. A new view of the tree of life. *Nature Microbiology* 2016; **1**: 1–6.
17. Hyatt D, Chen G-L, LoCascio PF, Land ML, Larimer FW, Hauser LJ. Prodigal: prokaryotic gene recognition and translation initiation site identification. *BMC Bioinformatics* 2010; **11**: 119.
18. Edgar RC. MUSCLE: multiple sequence alignment with high accuracy and high throughput. *Nucleic Acids Res* 2004; **32**: 1792–1797.
19. Capella-Gutiérrez S, Silla-Martínez JM, Gabaldón T. trimAl: a tool for automated alignment trimming in large-scale phylogenetic analyses. *Bioinformatics* 2009; **25**: 1972–1973.
20. Graham ED, Heidelberg JF, Tully BJ. BinSanity: unsupervised clustering of environmental microbial assemblies using coverage and affinity propagation. *PeerJ* 2017; **5**: e3035.
21. Price MN, Dehal PS, Arkin AP. FastTree 2 – Approximately Maximum-Likelihood Trees for Large Alignments. *PLOS ONE* 2010; **5**: e9490.
22. Letunic I, Bork P. Interactive Tree Of Life (iTOL) v4: recent updates and new developments. *Nucleic Acids Research* 2019; **47**: 256–259.
23. Chafee M, Fernàndez-Guerra A, Buttigieg PL, Gerdts G, Eren AM, Teeling H, et al. Recurrent patterns of microdiversity in a temperate coastal marine environment. *ISME J* 2018; **12**: 237–252.
24. Eren AM, Morrison HG, Lescault PJ, Reveillaud J, Vineis JH, Sogin ML. Minimum entropy decomposition: Unsupervised oligotyping for sensitive partitioning of high-throughput marker gene sequences. *ISME J* 2015; **9**: 968–979.

25. Orellana LH, Francis TB, Ferraro M, Hehemann J-H, Fuchs BM, Amann RI. Verrucomicrobiota are specialist consumers of sulfated methyl pentoses during diatom blooms. *ISME J* 2021; 1–12.
