# Supplementary material for: Niche partitioning of the ubiquitous and ecologically relevant NS5 marine group
Source: ISME J. 2022 Feb 15;16(6):1570–82. doi: 10.1038/s41396-022-01209-8 (PMC9122927; doi:10.1038/s41396-022-01209-8)
Supplement: Supplementary file 2 — Supplementary figures all [file 41396_2022_1209_MOESM2_ESM.pdf]

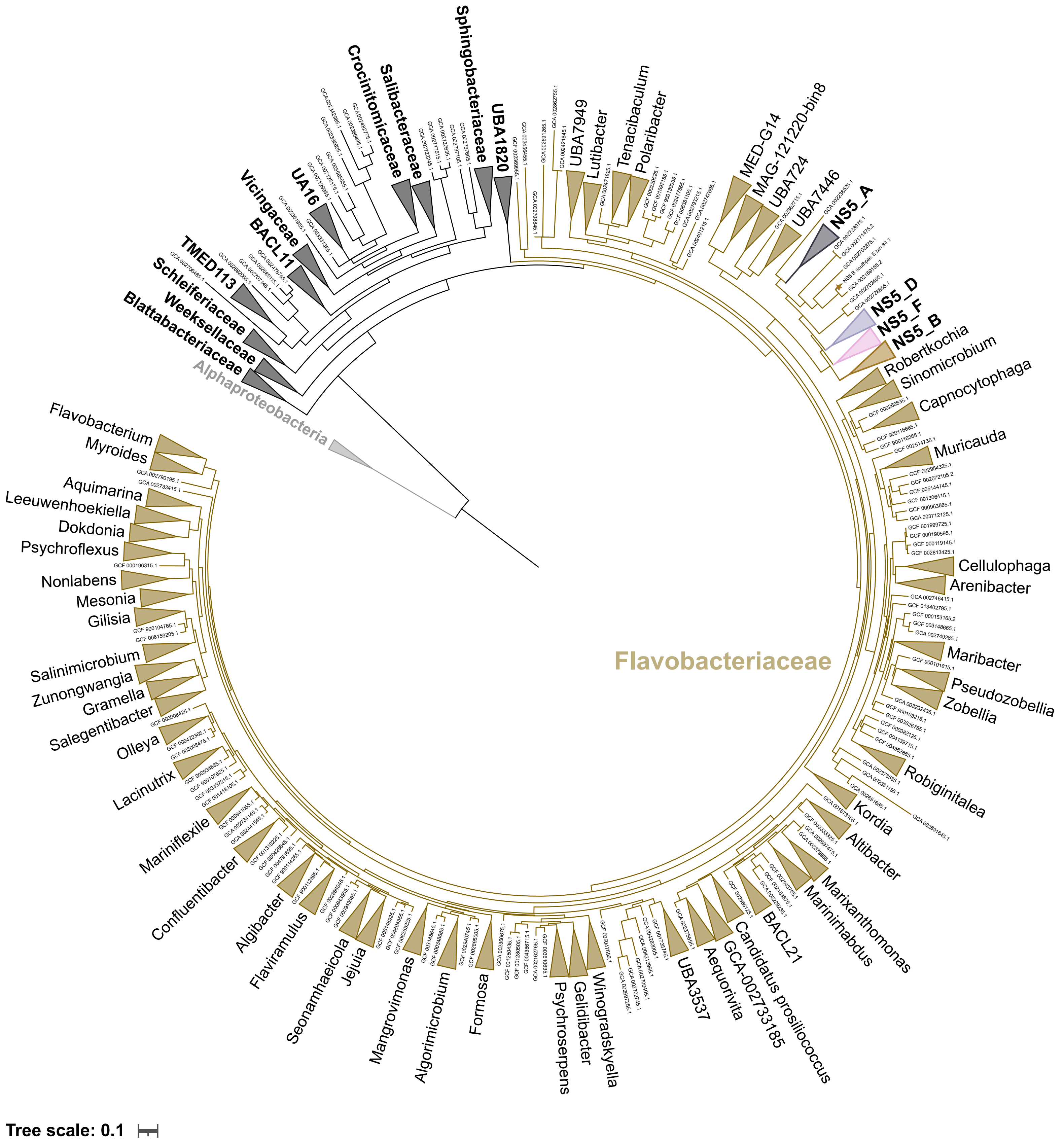

Supplementary Figure S1. Ribosomal protein tree of the Flavobacteriia class. The tree was constructed using a concatenated alignment of 16 ribosomal proteins from 1275 complete genome assemblies from RefSeq along with the 35 species-representaive NS5 MAG sequences. One species from NS5_B, indicated with a *, was positioned outside of the gens-level cluster.

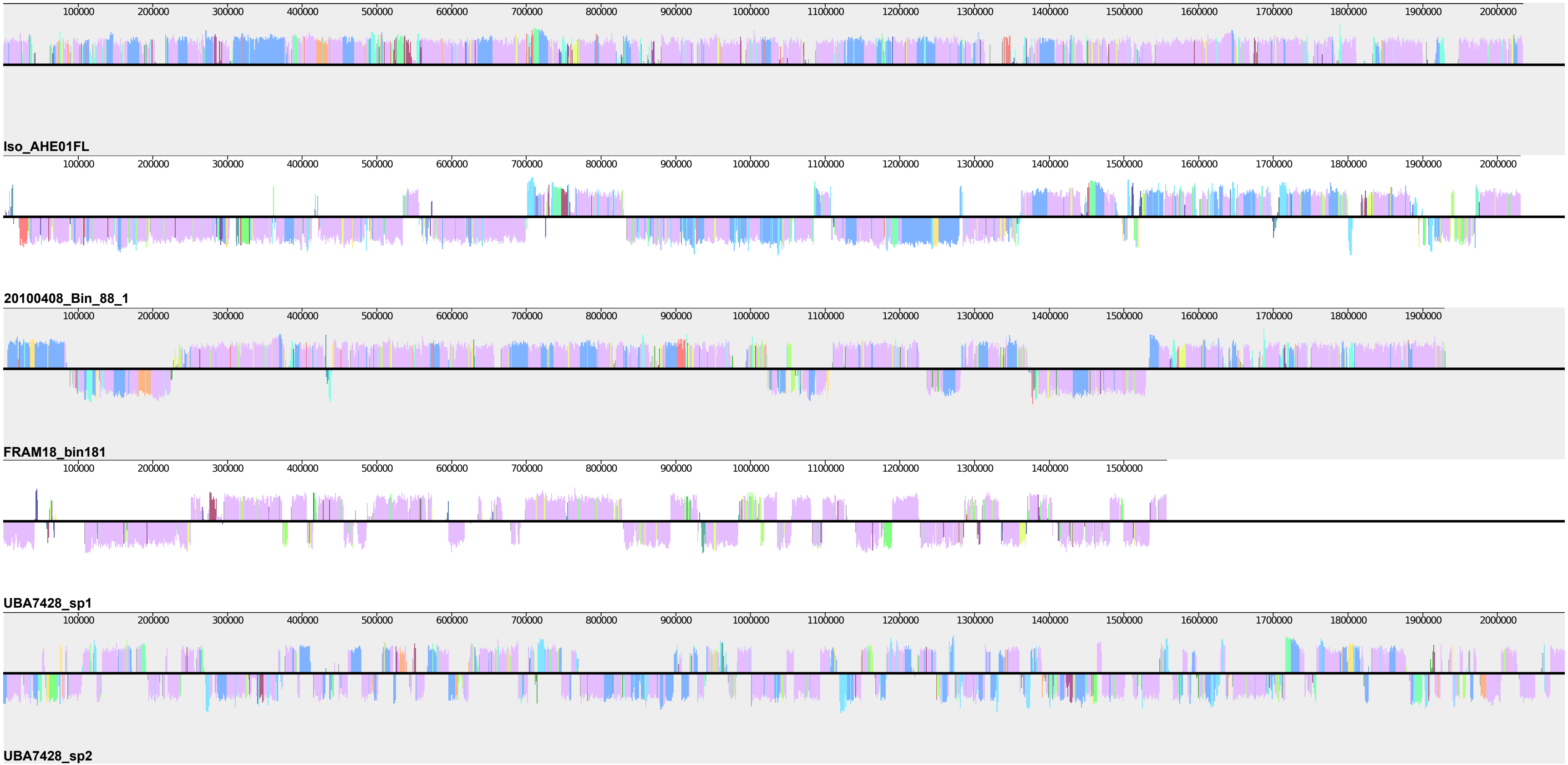

Supplementary Figure S2. Genome alignment and conserved syntenic block identification for species-representatives in NS5_F against the isolate genome, Iso_AHE01FL. Alignment was performed using the progressiveMauve aligner in the Mauve program with default settings. The colours represent conserved syntenic gene blocks, with the same colour across different genomes indicating a shared block. The regions shared by all speices-representatives are coloured in mauve.

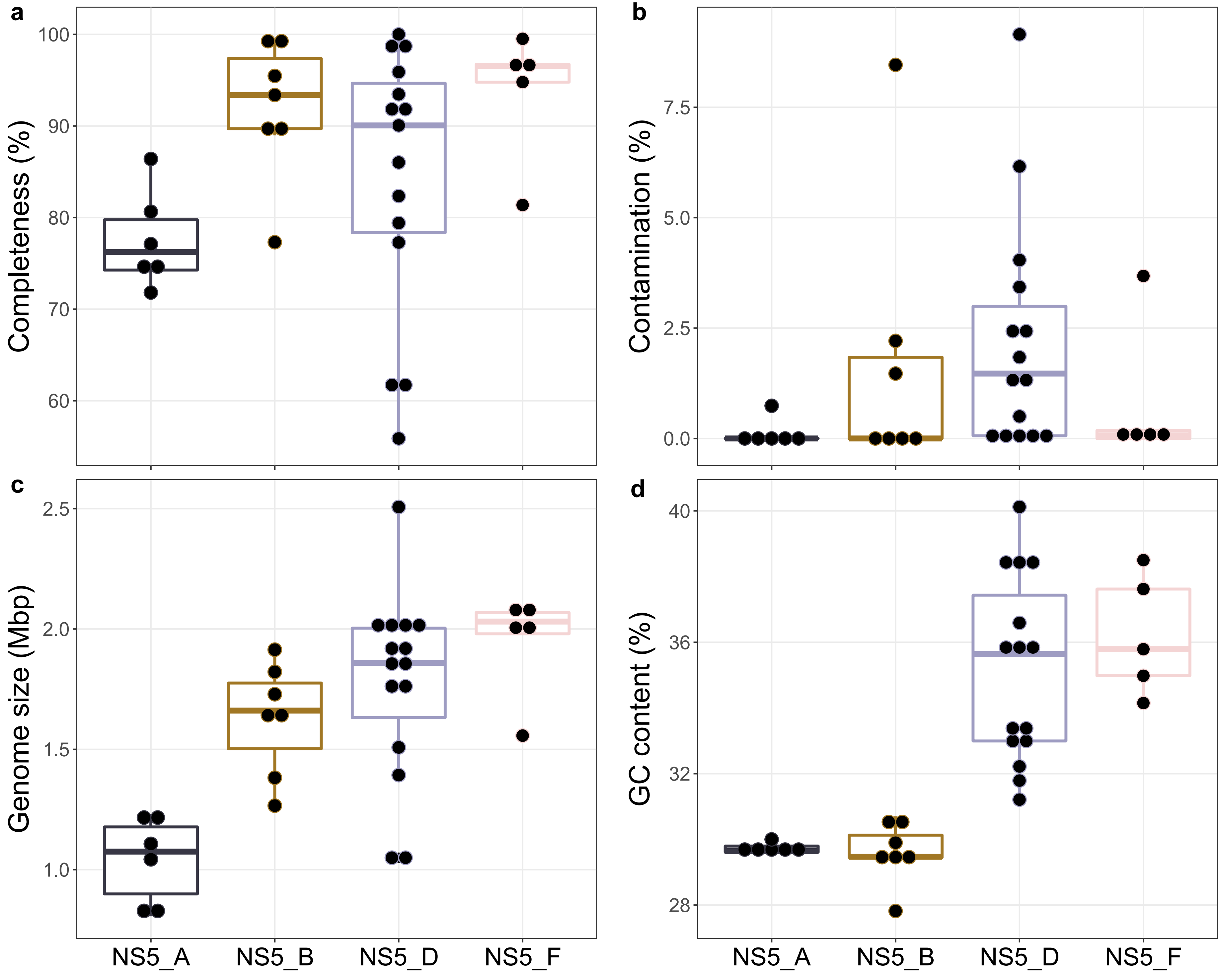

Supplementary Figure S3. Summary statistics of species-representative MAGs. All values were determined using CheckM.

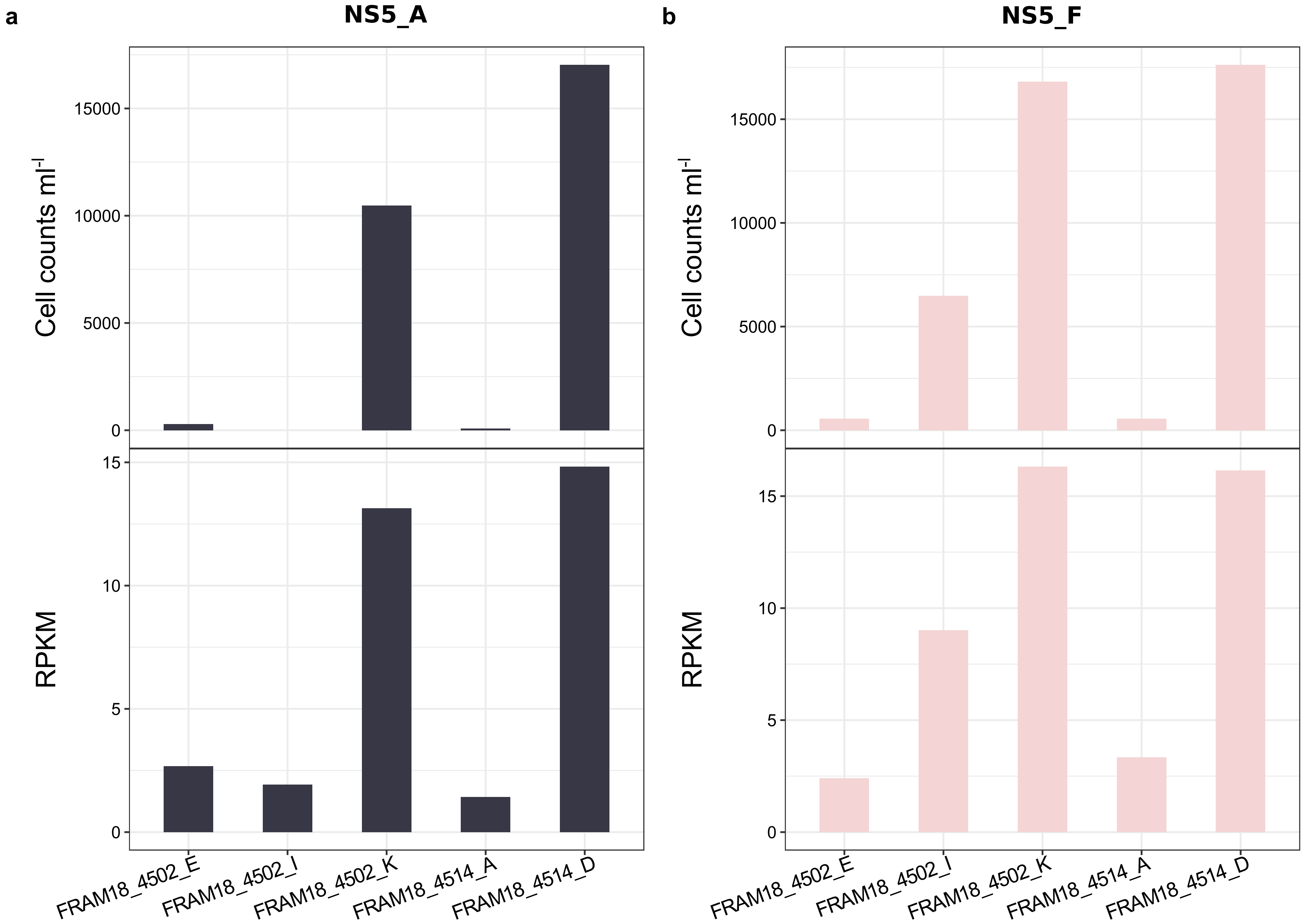

Supplementary Figure S4. Comparison of absolute cell counts and RPKM values from surface seawater samples, 0.2 - 3 µm fraction, taken in the Fram Strait region. Cell counts were derived from CARD-FISH analysis, where cells containing the group-specific probe signal and a nucleic acid stain were enumerated. RPKM values were based on read recruitment from sample metagenomes using BBMap with a 99% identity threshold. Samples used for this analysis were derived from [26], with sample names being maintained for comparison.

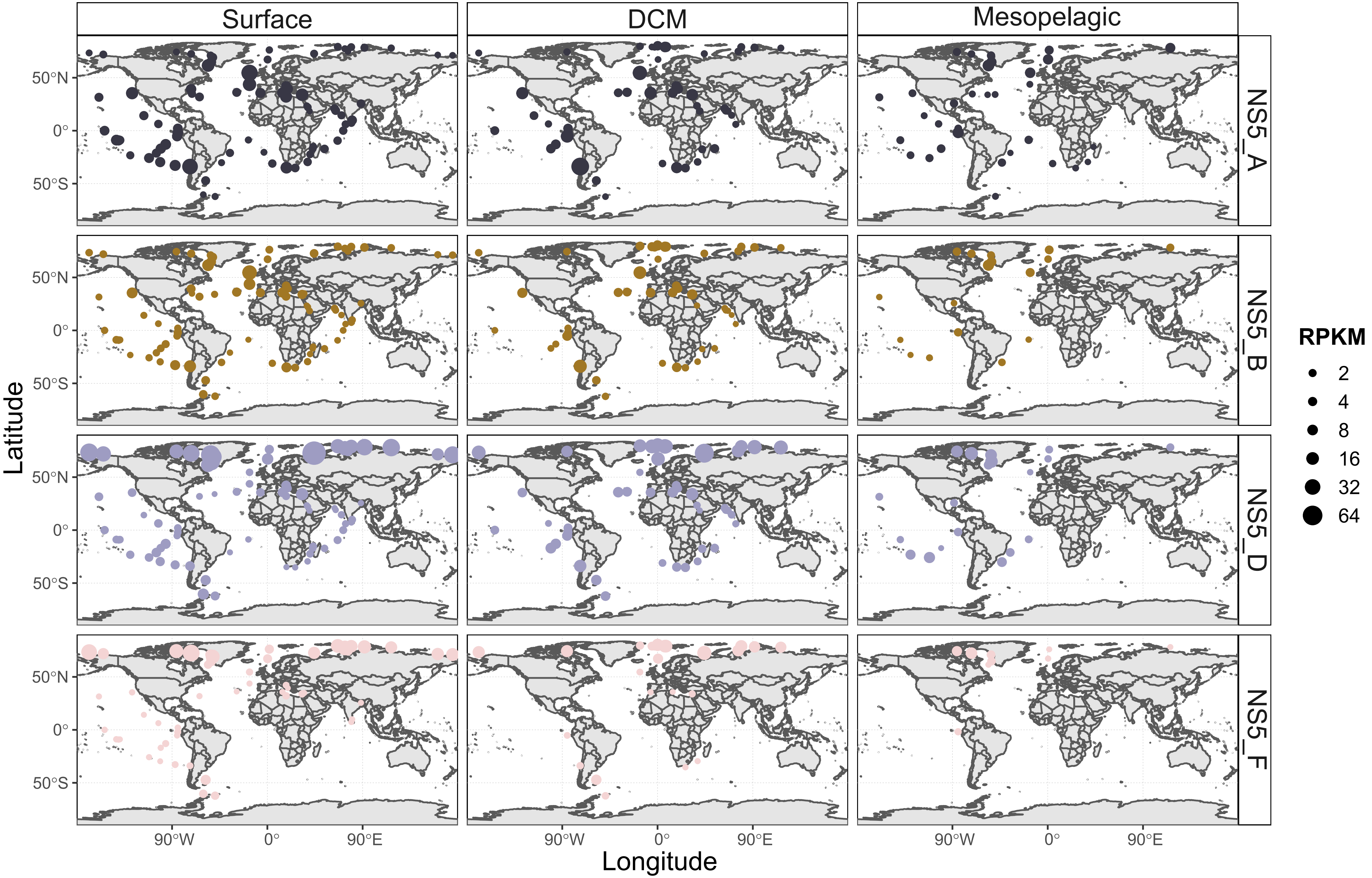

Supplementary Figure S5. Global distribution of NS5 genera from the surface to mesopelagic layer. Distribution was determined by read recruitment from TaraOceans metagenomic samples to each species-representative MAG using BBMap with a 99% identity threshold cut-off. The number of mapped reads was converted to RPKM values and a minimum threshold of 0.25 RPKM was applied, which ensured a minimum coverage of 40%. Genus RPKM values were obtained by summing the respective species' RPKM values for each sample.

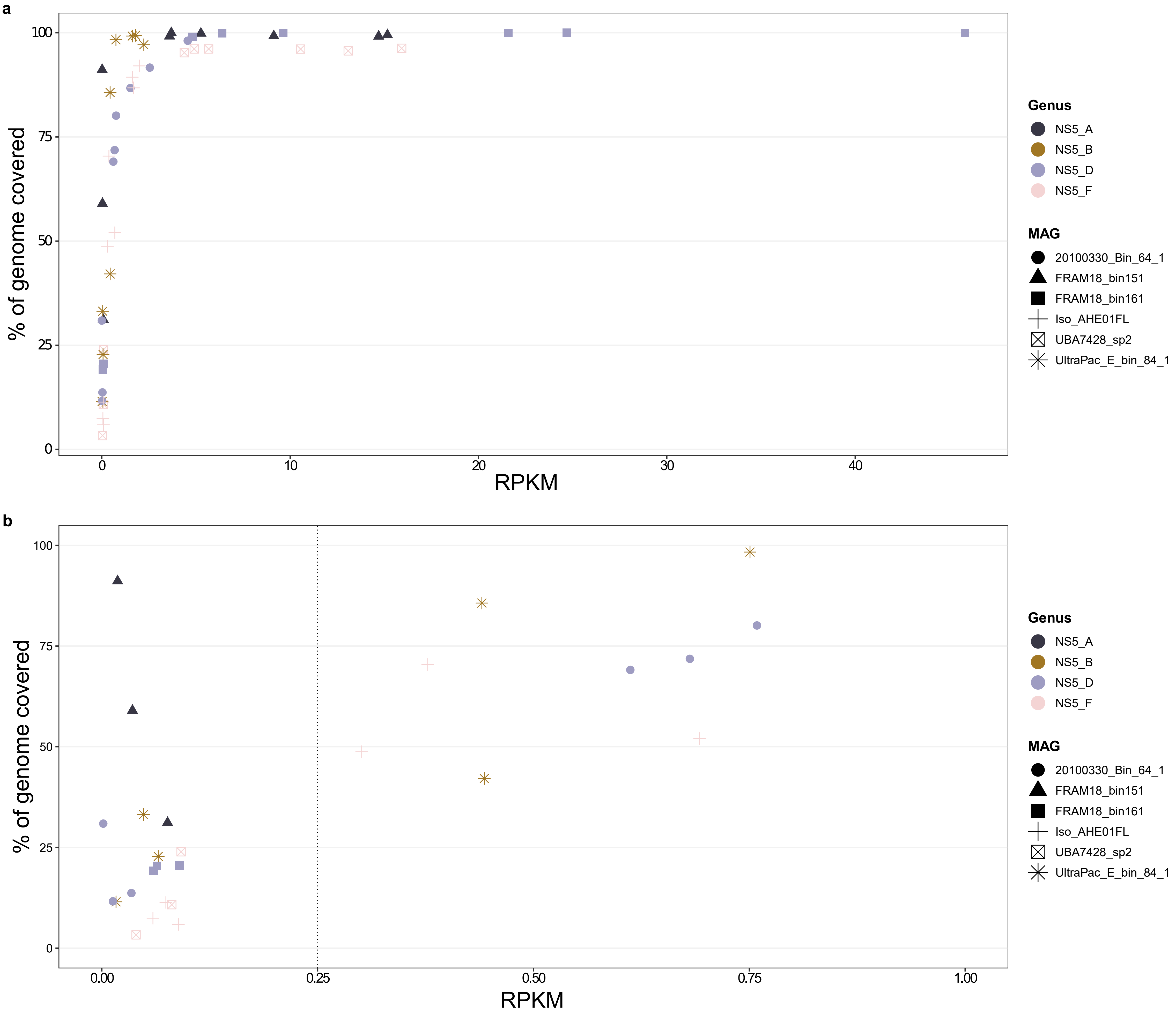

Supplementary Figure S6. RPKM values compared to genome coverage from the mapping of reads from Tara Oceans metagenome samples to selected NS5 species representatives. Representatives were selected based on exhibiting a large range in RPKM values across samples, and the samples in which they exhibited high, medium and low RPKM values were chosen for visualisation in this figure. Read recruitment was performed using BBMap with an identity threshold of 99%.

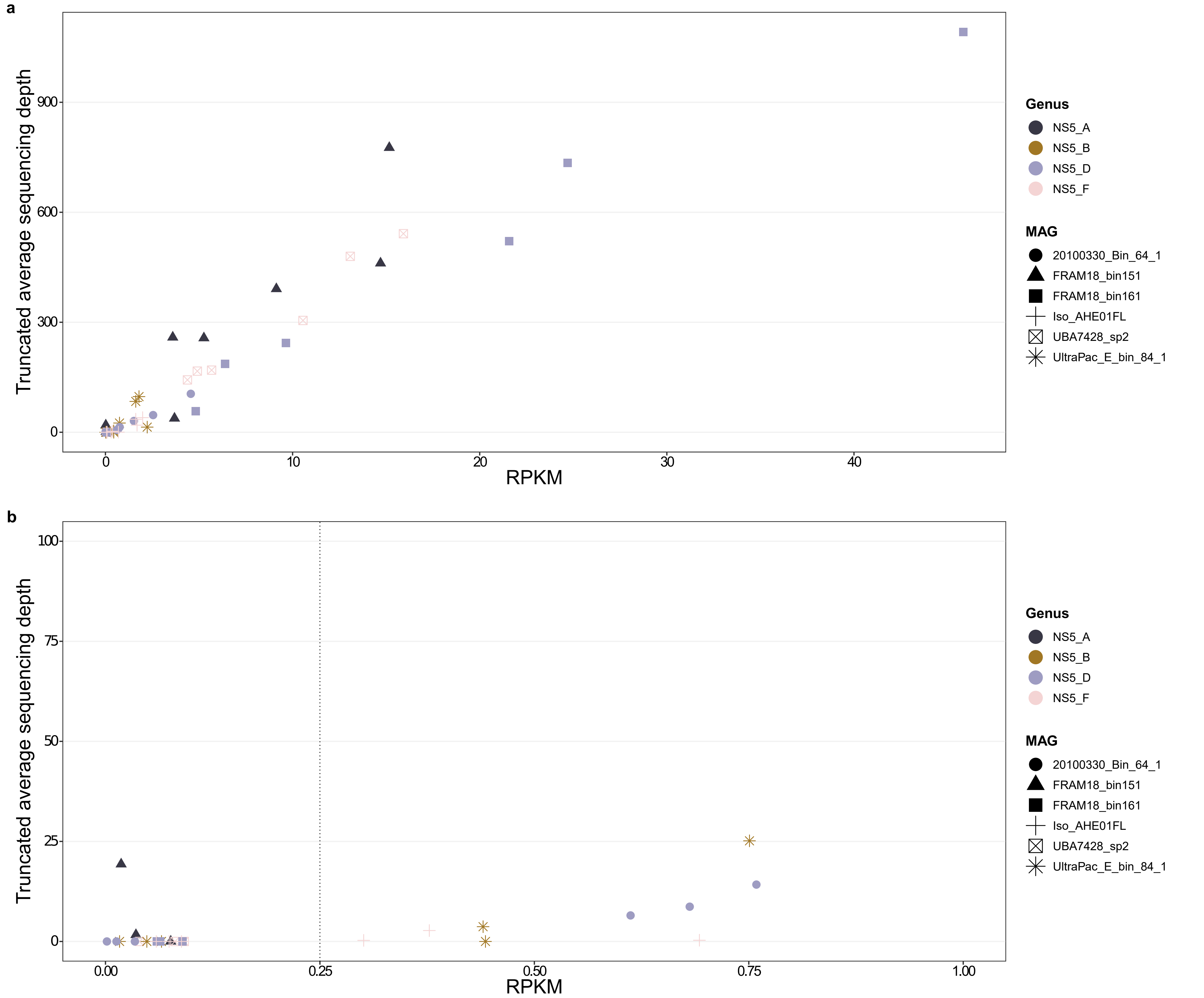

Supplementary Figure S7. RPKM values compared to truncated average depth from the mapping of reads from Tara Oceans metagenome samples to selected NS5 species representatives. Representatives were selected based on exhibiting a large range in RPKM values across samples, and the samples in which they exhibited high, medium and low RPKM values were chosen for visualisation in this figure. Read recruitment was performed using BBMap with an identity threshold of 99%. Truncated average depth values were calculated according to [61].

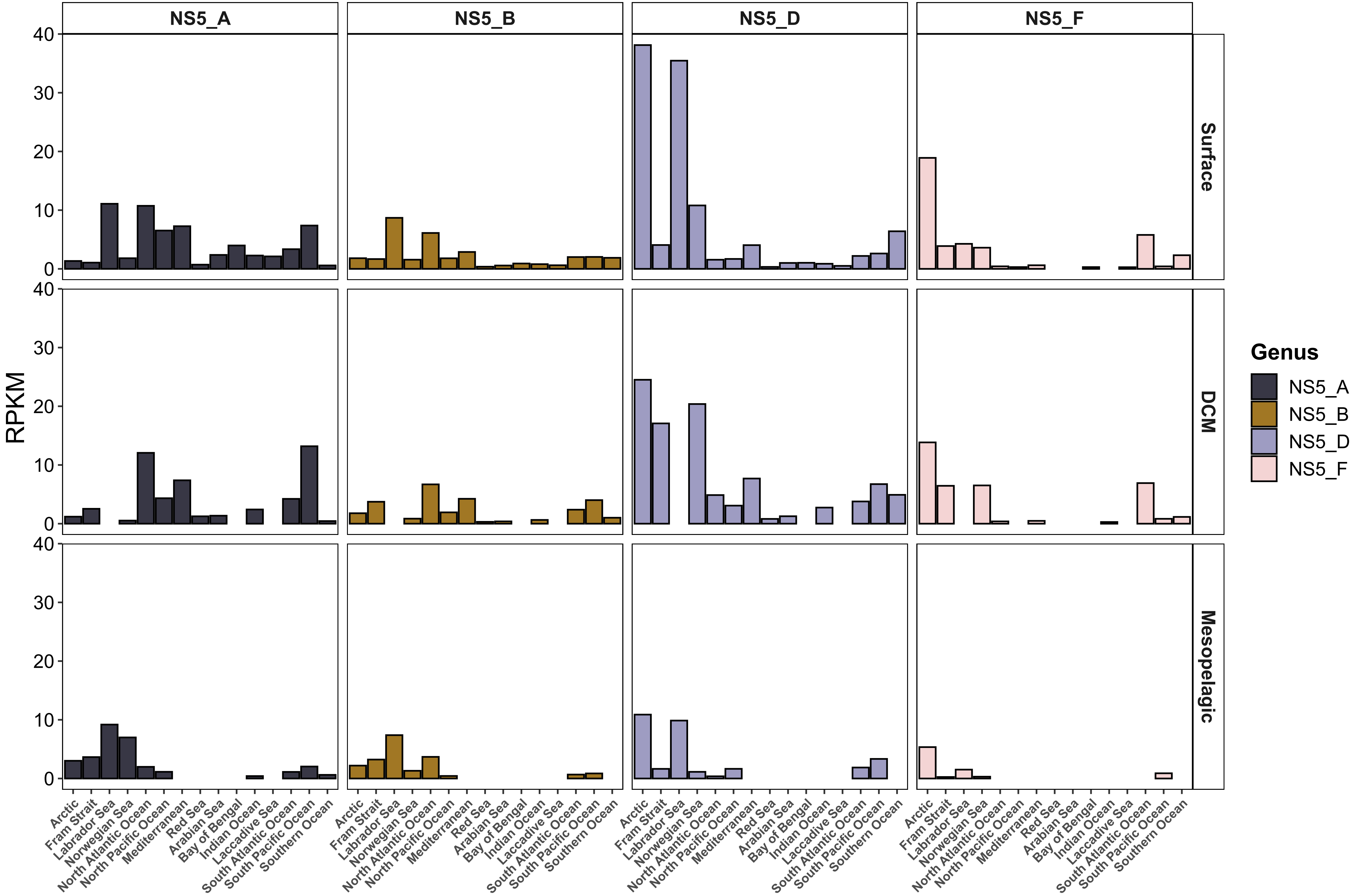

Supplementary Figure S8. Distribution of NS5 genera across oceanic regions. Reads were recruited from Tara Oceans metagenomes to each species-representative MAG using BBMap with a 99% identity threshold cut-off and subsequently converted to RPKM values. Genus-level RPKM values were derived from the sum of the respective species' RPKM values in each sample. Tara Oceans metagenomes were designated into oceanic regions based on latitude and longitude. RPKM value for each genus in each region was derived from the average RPKM values of the genus in all samples of that region.

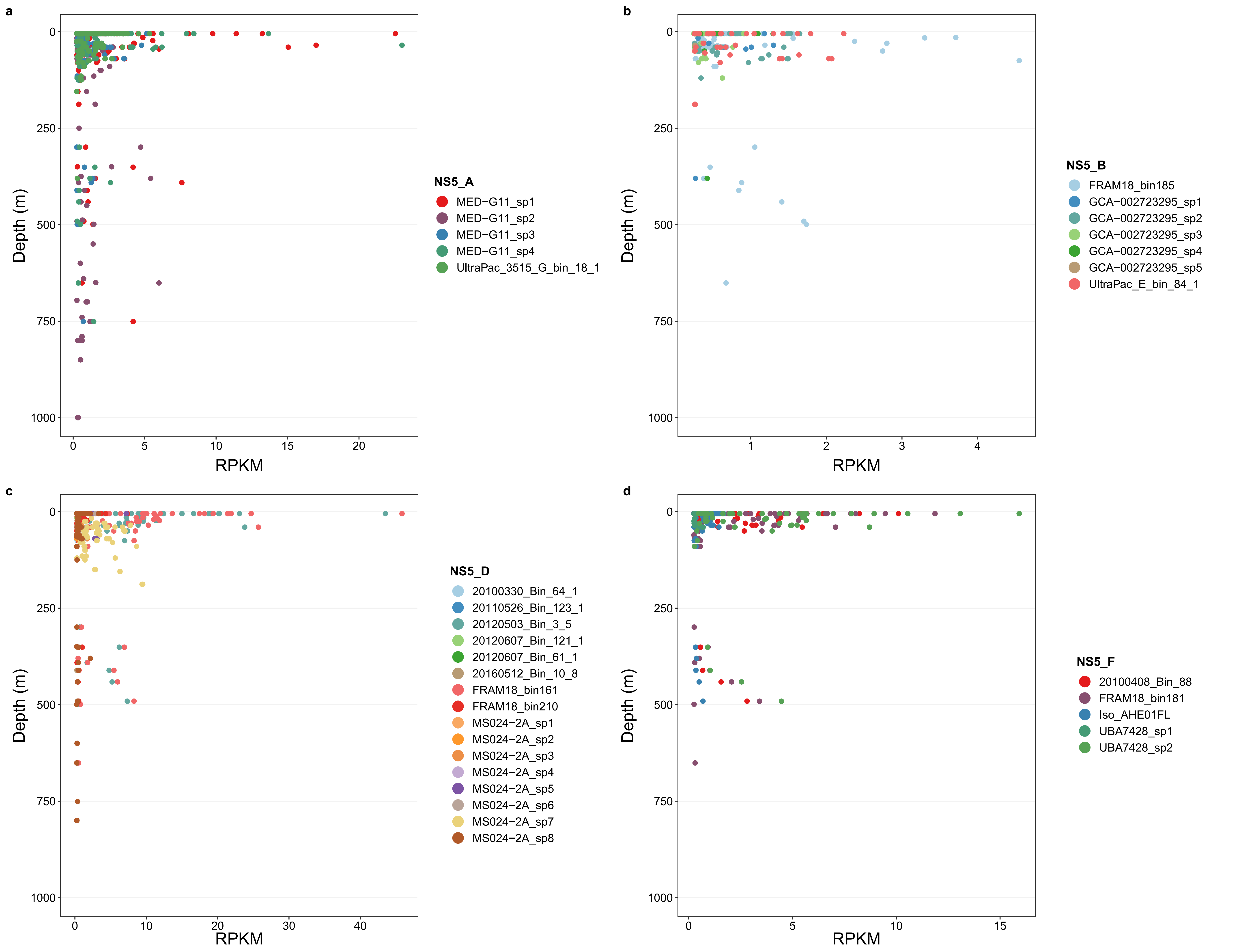

Supplementary Figure S9. RPKM values of NS5 species representatives in relation to depth across Tara Oceans samples. RPKM values were determined through read recruitment from Tara Oceans metagenomes using BBMap with a 99% identity threshold.

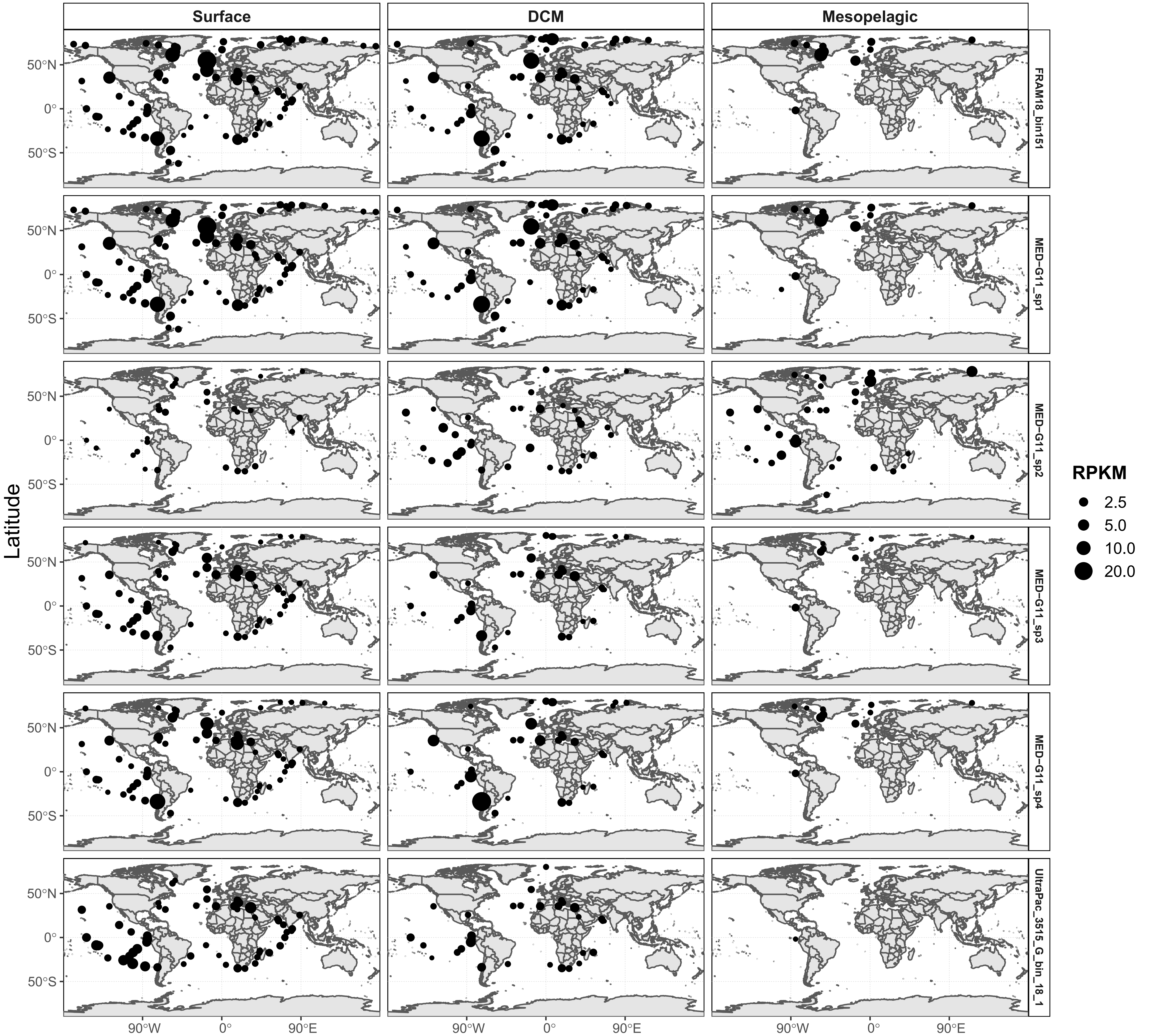

Supplementary Figure S10. Global distribution of the species-representatives within NS5_A from the surface to mesopelagic layer. RPKM values were determined from read recruitment of Tara Oceans metagenomic reads to each representative using BBMap with a 99% identity threshold cut-off. Resulting RPKM values <0.25 were excluded from the plot, which ensured a minimum genome coverage of 40%.

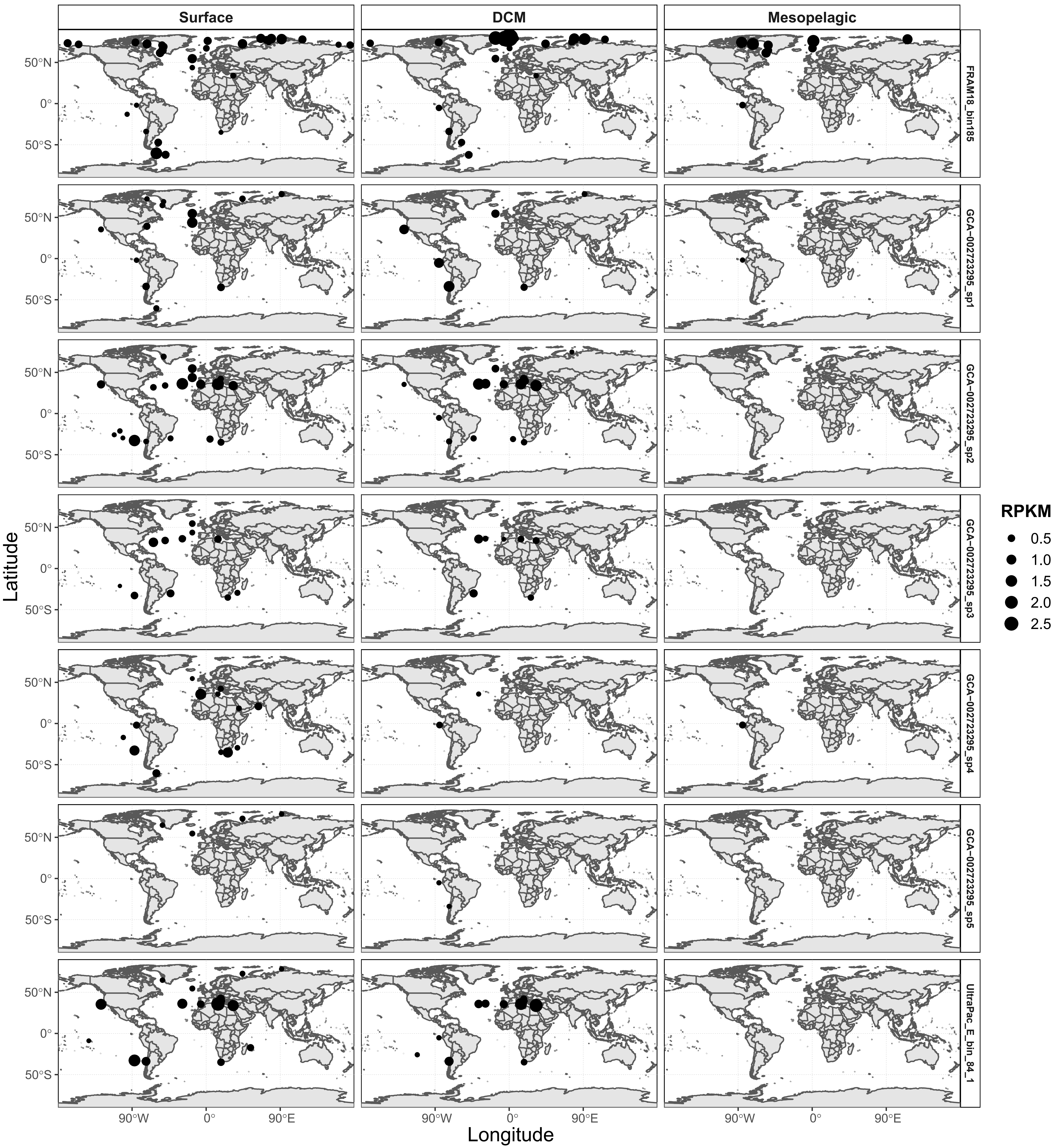

Supplementary Figure S11. Global distribution of the species-representatives within NS5_B from the surface to mesopelagic layer. RPKM values were determined from read recruitment of Tara Oceans metagenomic reads to each representative using BBMap with a 99% identity threshold cut-off. Resulting RPKM values <0.25 were excluded from the plot, which ensured a minimum genome coverage of 40%.

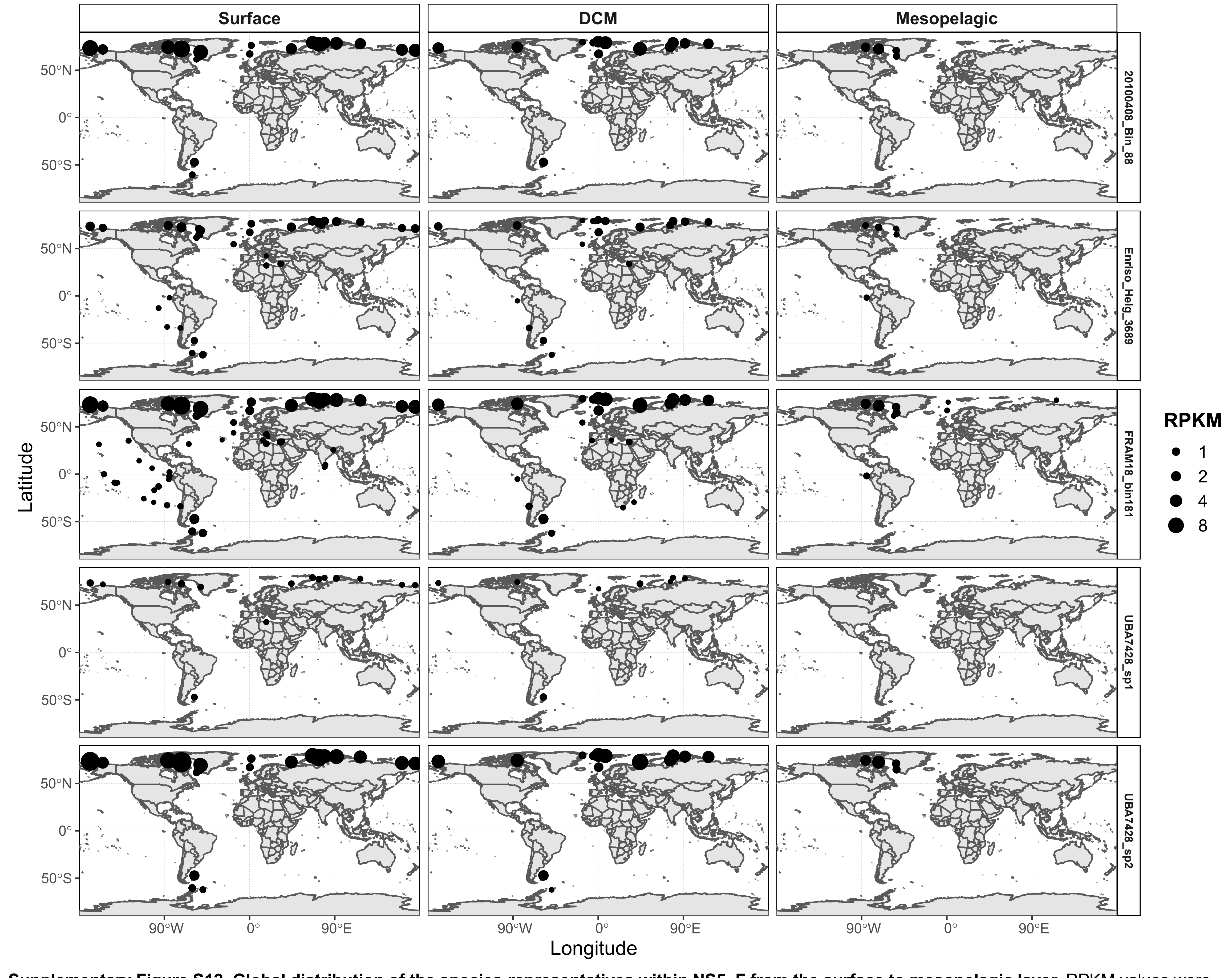

Supplementary Figure S12. Global distribution of the species-representatives within NS5_F from the surface to mesopelagic layer. RPKM values were determined from read recruitment of Tara Oceans metagenomic reads to each representative using BBMap with a 99% identity threshold cut-off. Resulting RPKM values <0.25 were excluded from the plot, which ensured a minimum genome coverage of 40%.

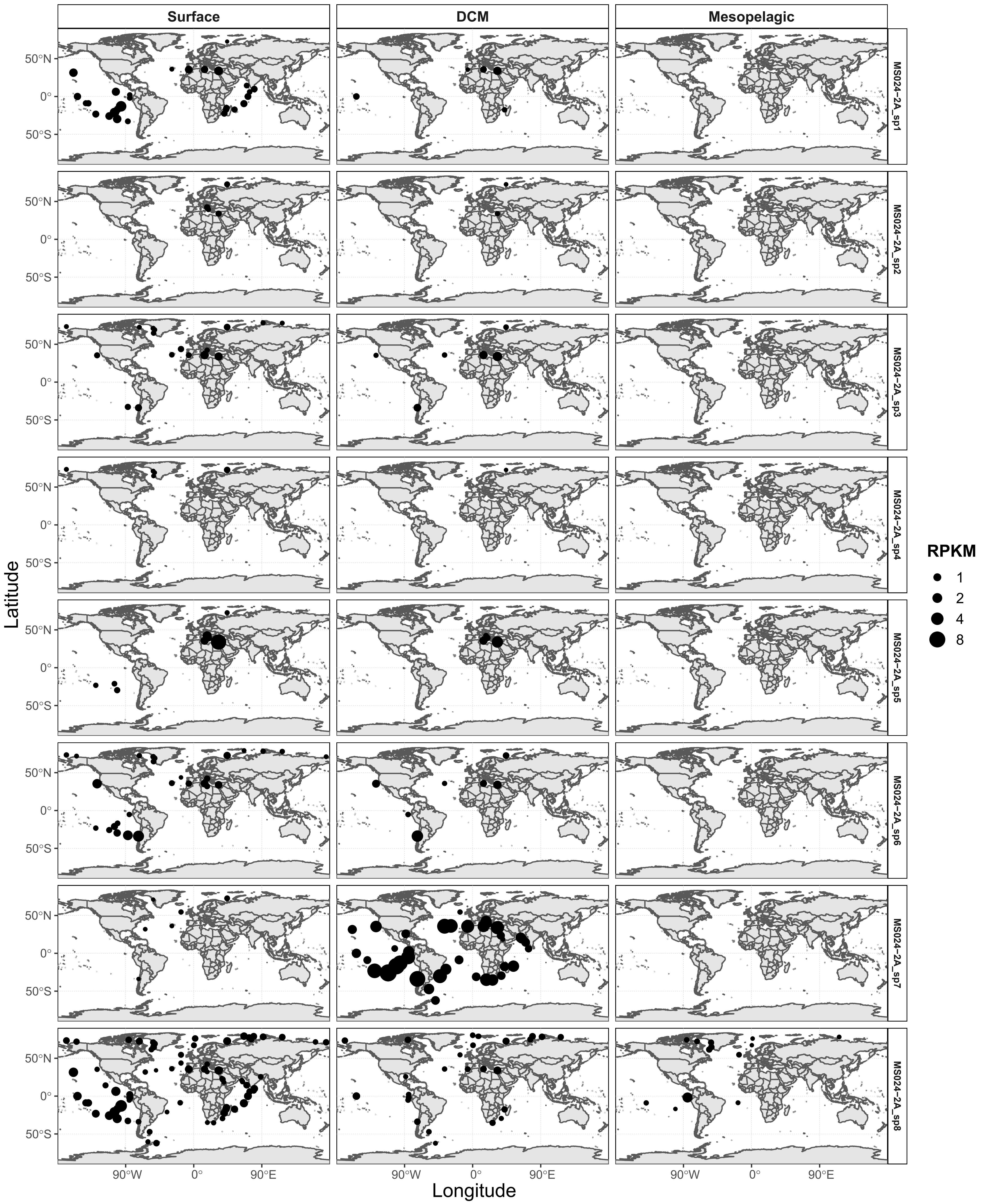

Supplementary Figure S13. Global distribution of the species-representatives within NS5_D from the surface to mesopelagic layer. RPKM values were determined from read recruitment of Tara Oceans metagenomic reads to each representative using BBMap with a 99% identity threshold cut-off. Resulting RPKM values <0.25 were excluded from the plot, which ensured a minimum genome coverage of 40%. Due to the large number of species in NS5_D, this plot includes only half, whilst the other half are visualised in Supplementary Figure S14.

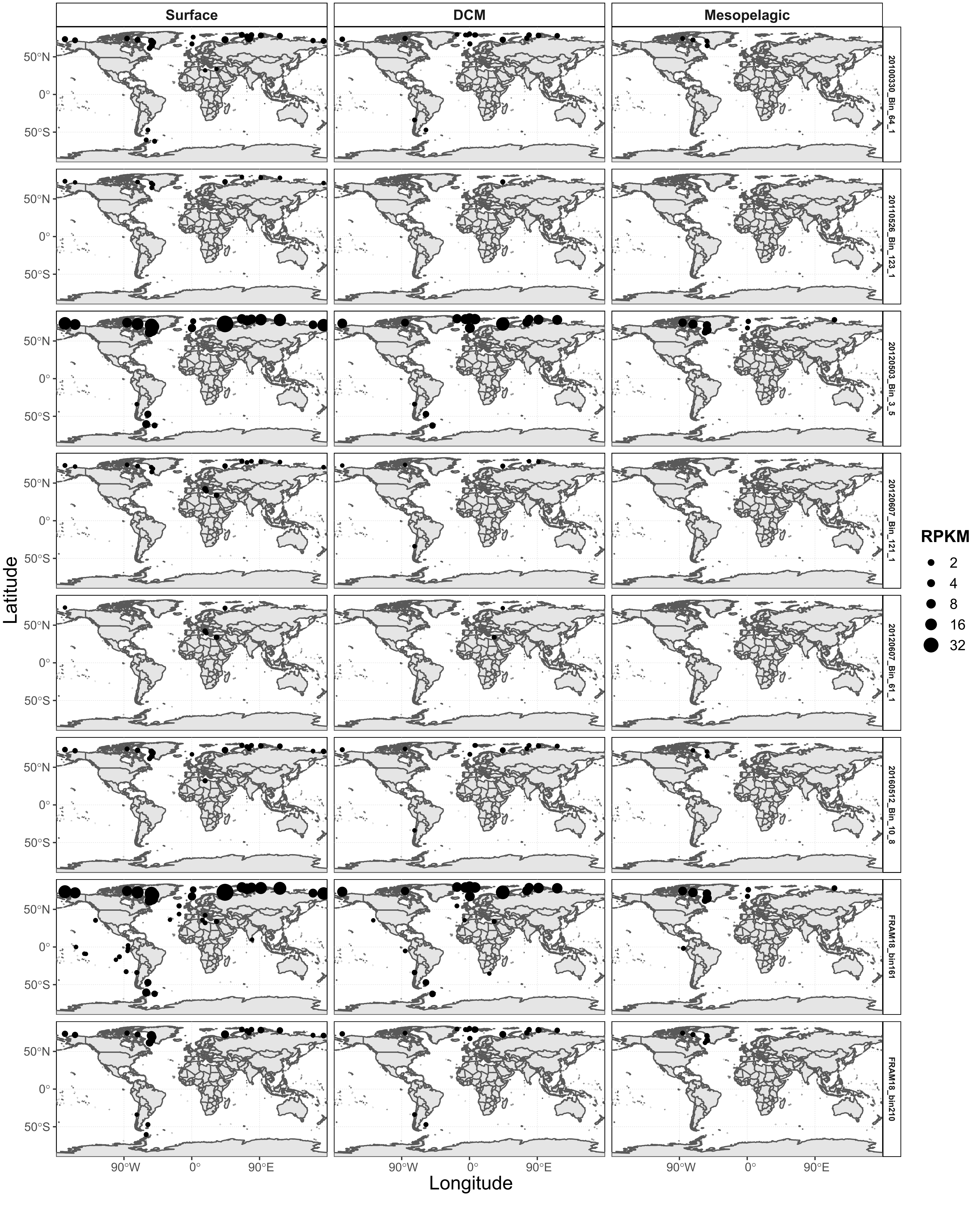

Supplementary Figure S14. Global distribution of the species-representatives within NS5_D from the surface to mesopelagic layer. RPKM values were determined from read recruitment of Tara Oceans metagenomic reads to each representative using BBMap with a 99% identity threshold cut-off. Resulting RPKM values <0.25 were excluded from the plot, which ensured a minimum genome coverage of 40%. Due to the large number of species in NS5_D, this plot includes only half, whilst the other half are visualised in Supplementary Figure S13.

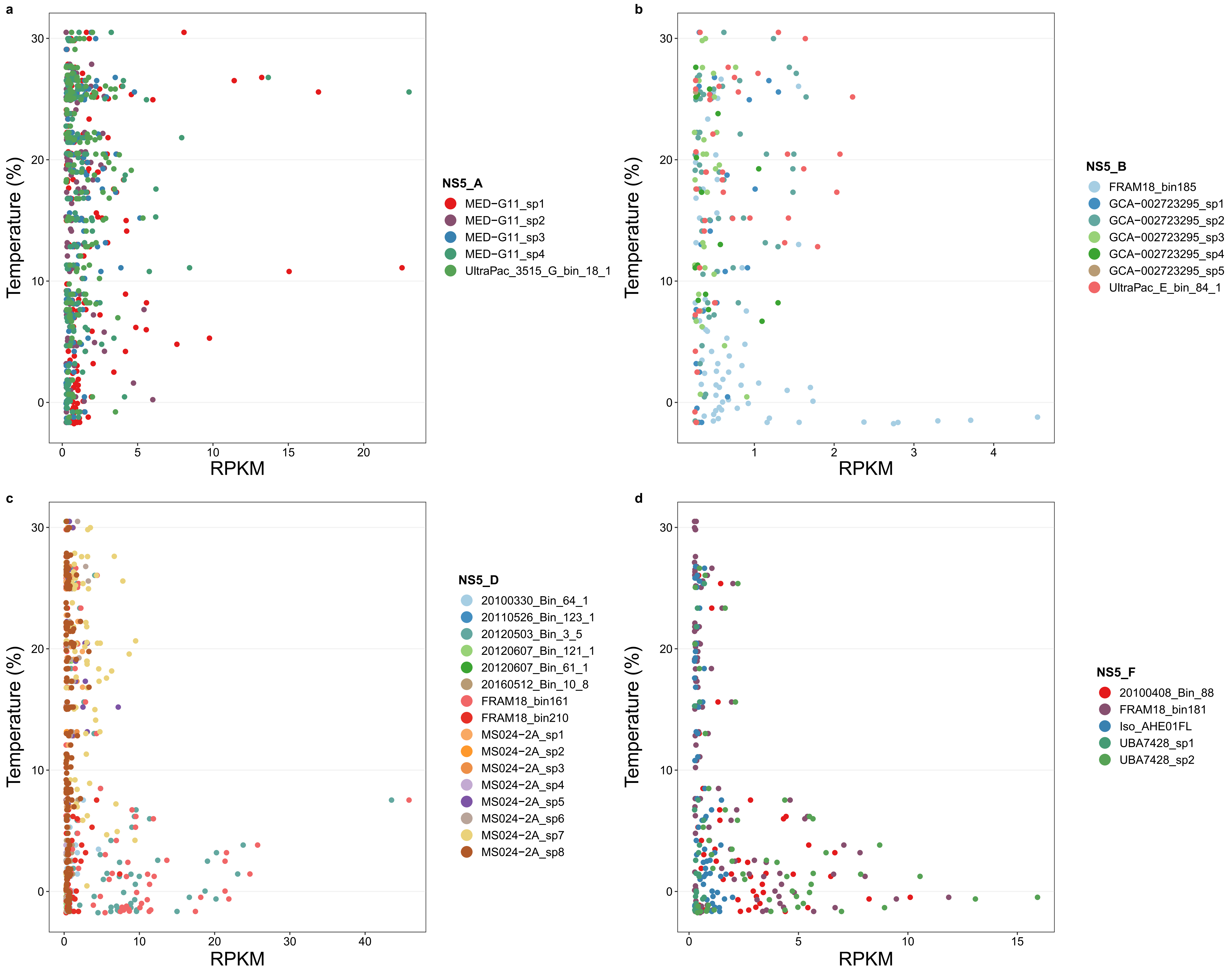

Supplementary Figure S15. RPKM values of NS5 species representatives in relation to temperature across Tara Oceans samples. RPKM values were determined through read recruitment from Tara Oceans metagenomes using BBMap with a 99% identity threshold.

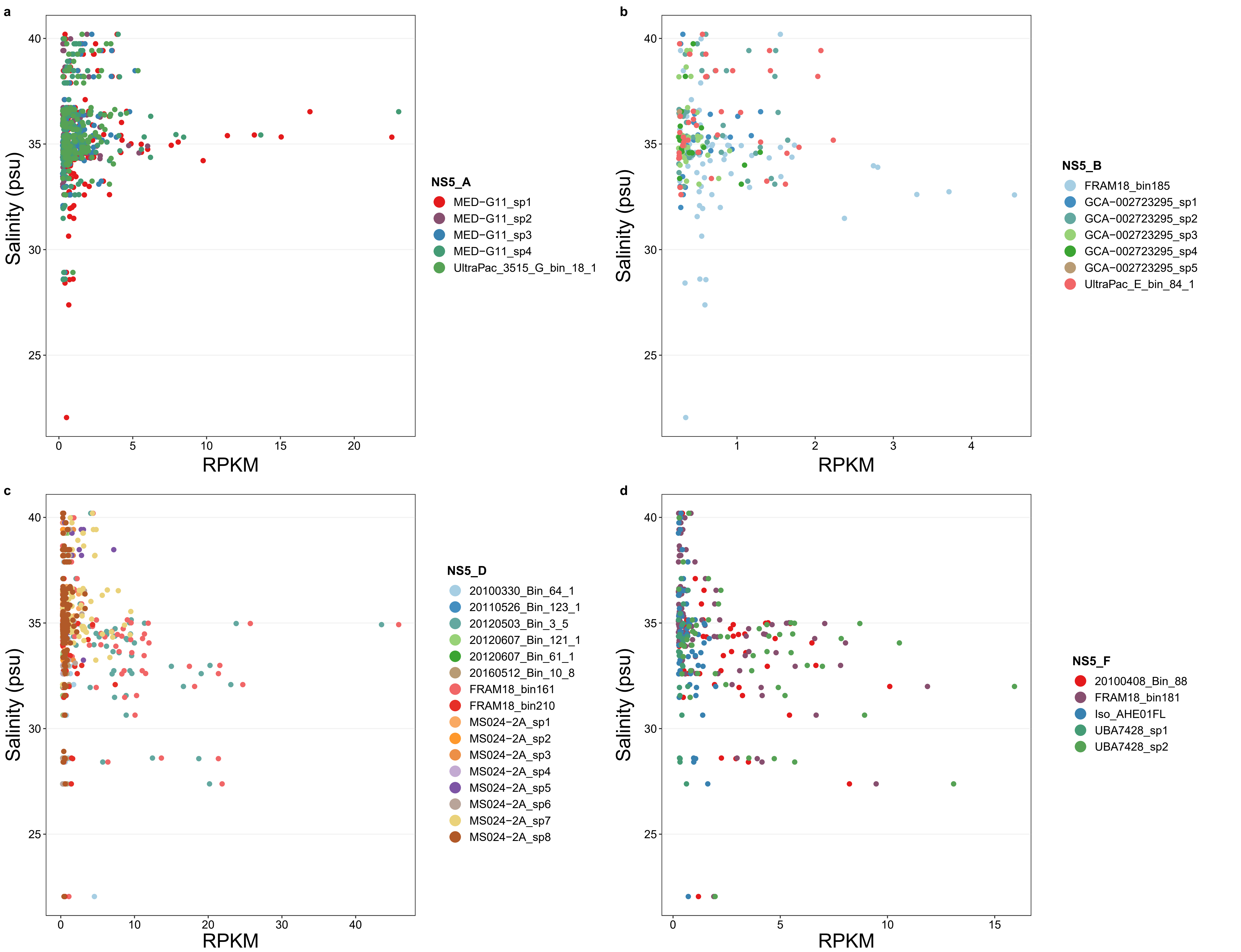

Supplementary Figure S16. RPKM values of NS5 species representatives in relation to salinity across Tara Oceans samples. RPKM values were determined through read recruitment from Tara Oceans metagenomes using BBMap with a 99% identity threshold.

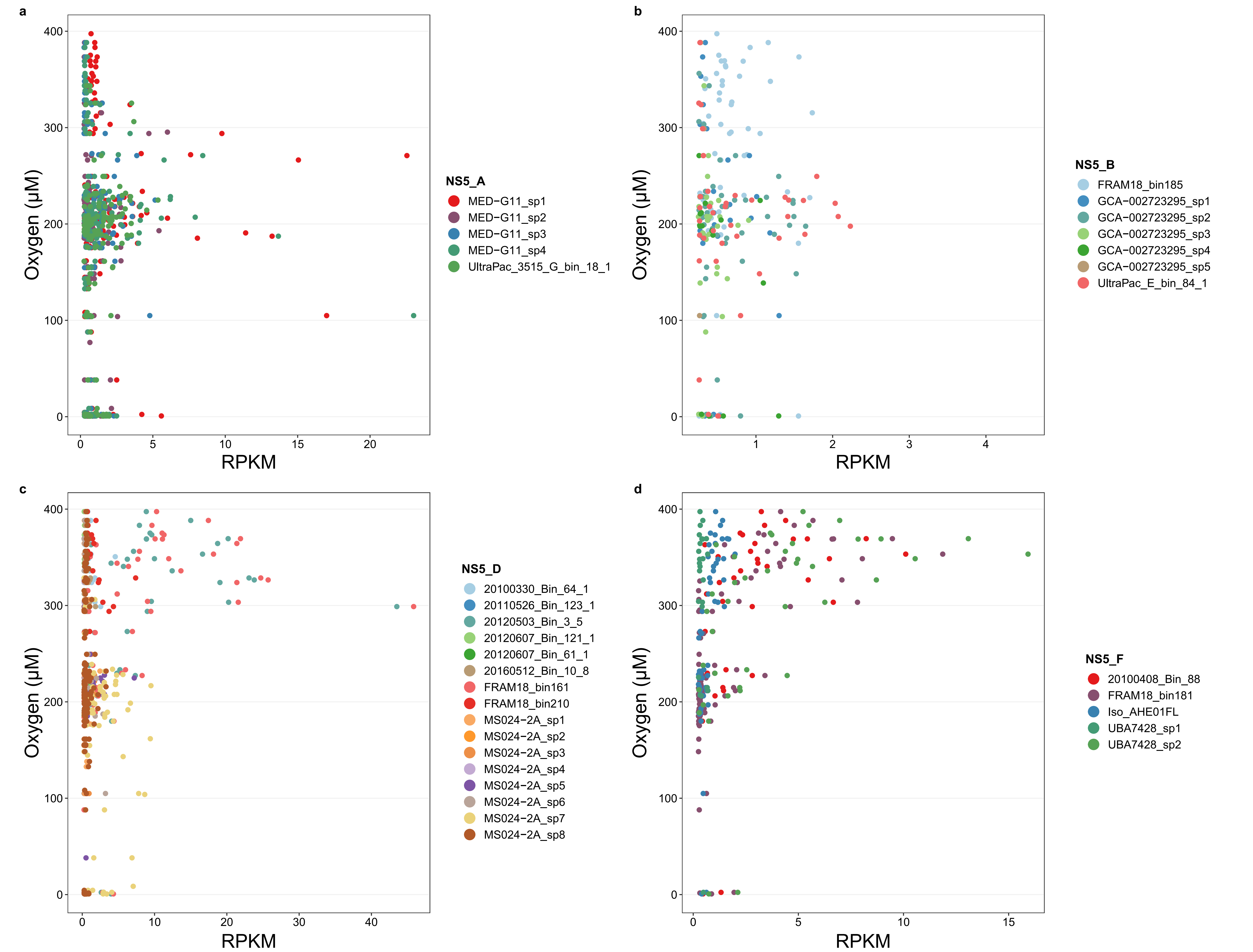

Supplementary Figure S17. RPKM values of NS5 species representatives in relation to oxygen across Tara Oceans samples. RPKM values were determined through read recruitment from Tara Oceans metagenomes using BBMap with a 99% identity threshold.

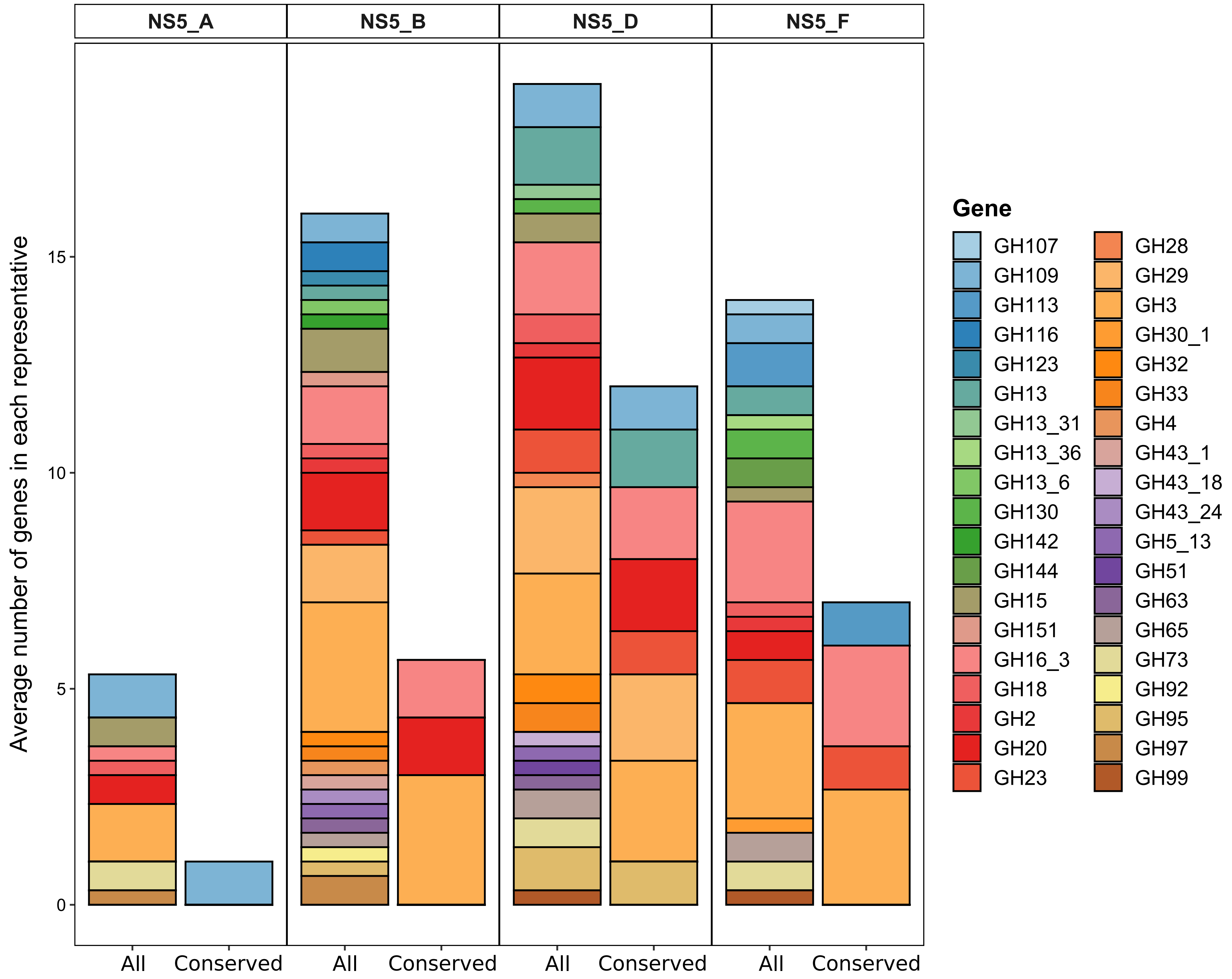

Supplementary Figure S18. Composition of conserved and non-conserved glycoside hydrolase gene family annotations from the three selected genus-representatives. The annotation of gene families was derived from agreements between HMMscan against the dbCAN v9 database and Diamond blastp search against the CAZy database. Genus values presented were derived from the average of the gene counts of the three selected genus-representatives. Conserved genes were required to be present in all three representatives.

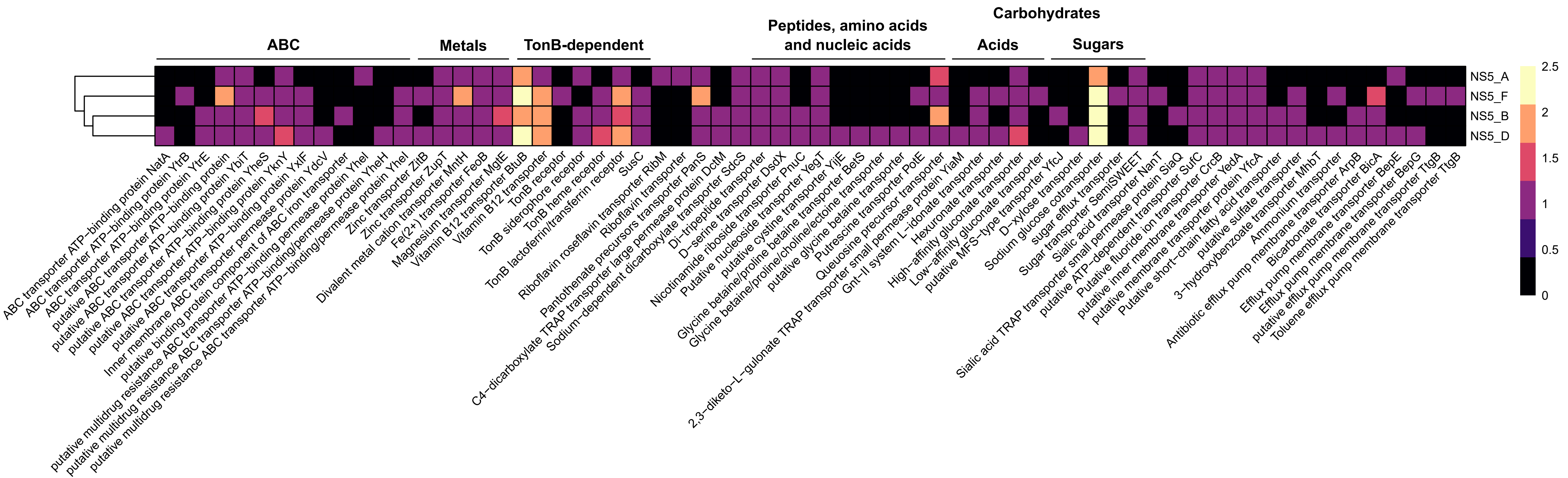

Supplementary Figure S19. Composition of transporter genes derived from the three selected genus-representative species. The gene number values shown are the average of the the three representatives from each genus. The annotation of transporter genes was performed with Prokka v1.14.6 [66].

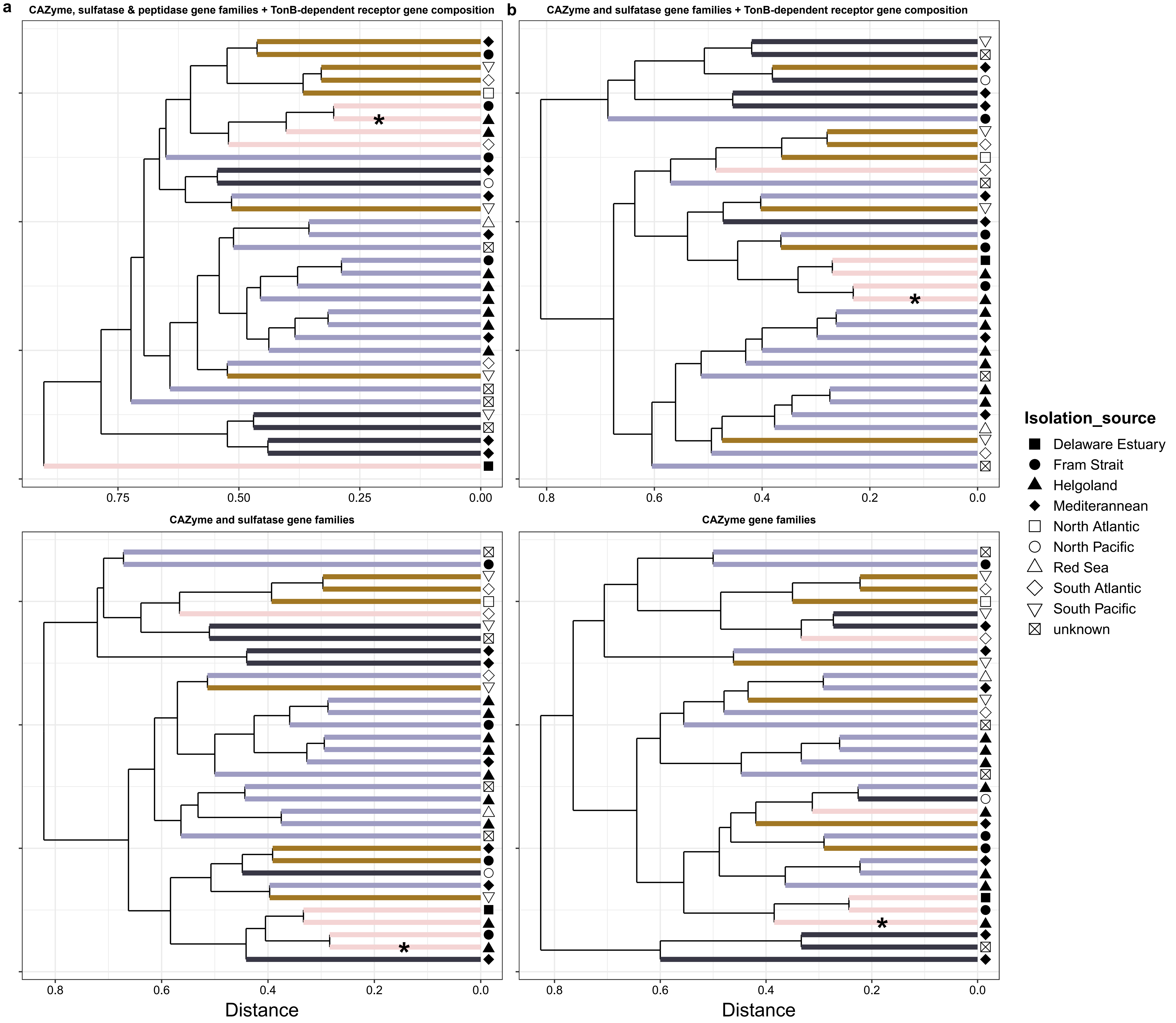

Supplementary Figure S20. Hierarchical clustering analysis of substrate utilisation gene composition of species-representative MAGs. Gene composition was converted to a Bray-Curtis dissimilarity matrix prior to clustering. **a)** Composition of CAZyme, sulfatase, peptidase and TonB-dependent transporter genes, **b)** Composition of CAZyme, sulfatase and TonB-dependent transporter genes, **c)** Composition of CAZyme and sulfatase genes, **d)** Composition of CAZyme genes. * indicates the isolate, iso_AHE01FL.

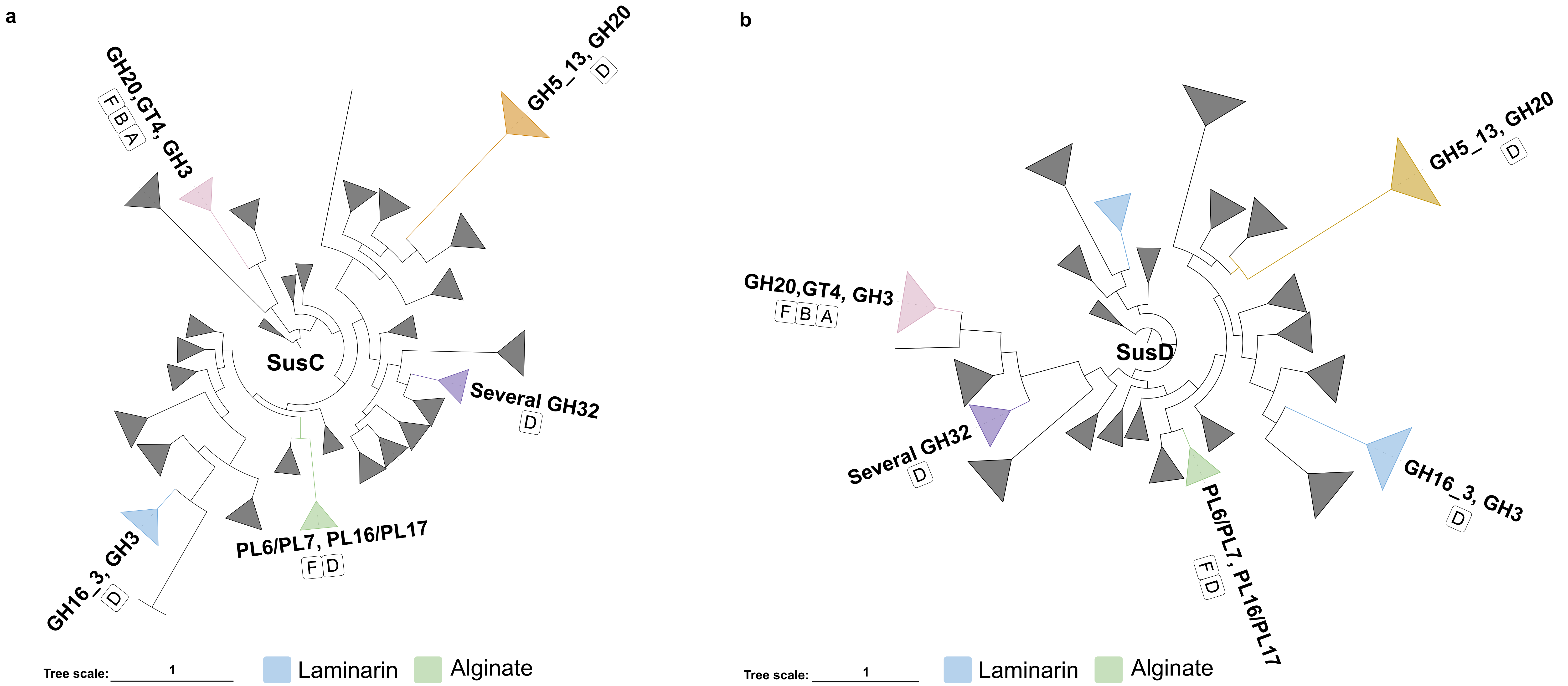

Supplementary Figure S21. Protein trees of SusC/SusD genes identified on polysaccharide utilisation loci of NS5 species representatives and other flavobacteria. a) Susc, **b)** SusD amino acid sequences from NS5 species along with those from previously published datasets of flavobacteria MAGs and cultured isolates were aligned used MAFFT L-INS-I and trees calculated using FastTree. Clusters that are coloured are those that include NS5-derived sequences and conserved gene colocalisations, indicated in the labels. The lettering underneath the cluster labels represent the genus affiliation of the NS5 species within that cluster. Blue branch containing GH16_3 and GH3 represents laminarin-targeting PULs whereas the green branch containing PL6/PL7 and PL16/PL17 represents alginate-targeting PULs.

Candidatus Marisimplicoccus framensis (NS5_A)

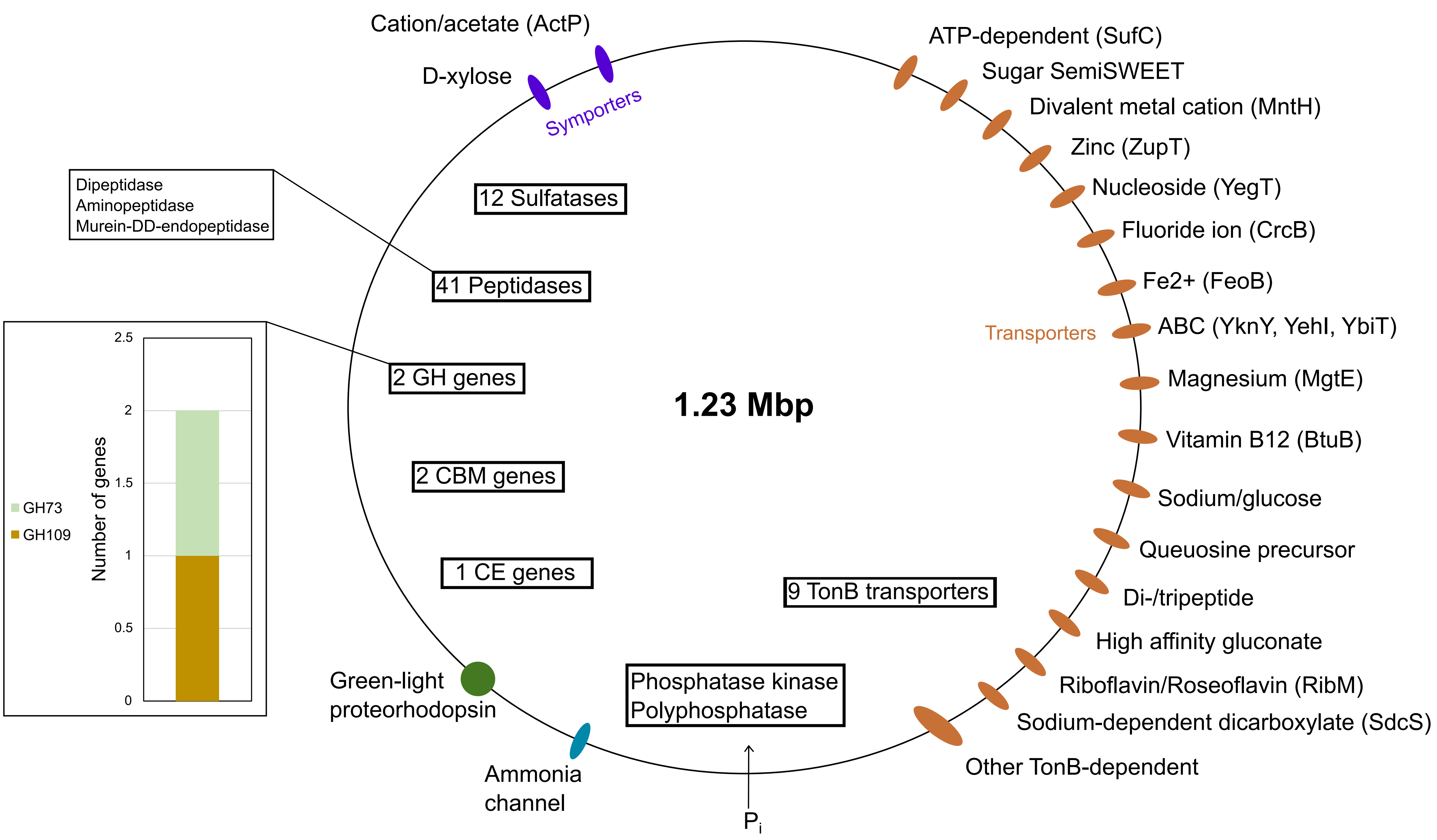

Supplementary Figure S22. Summary schematic of metabolism of type species *Candidatus* Marisimplicoccus framensis

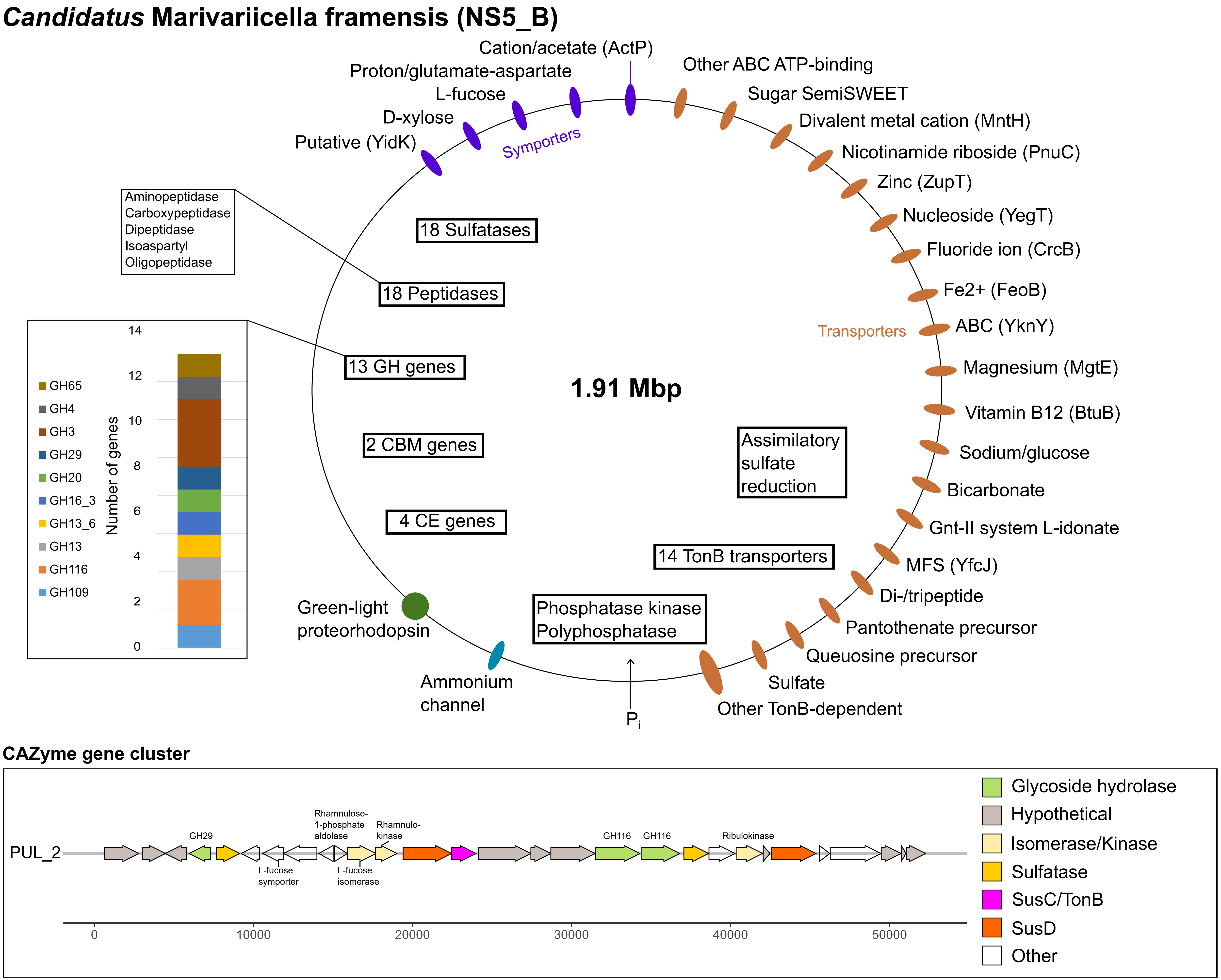

Supplementary Figure S23. Summary schematic of the metabolism of type species *Candidatus* Marivariicella framensis

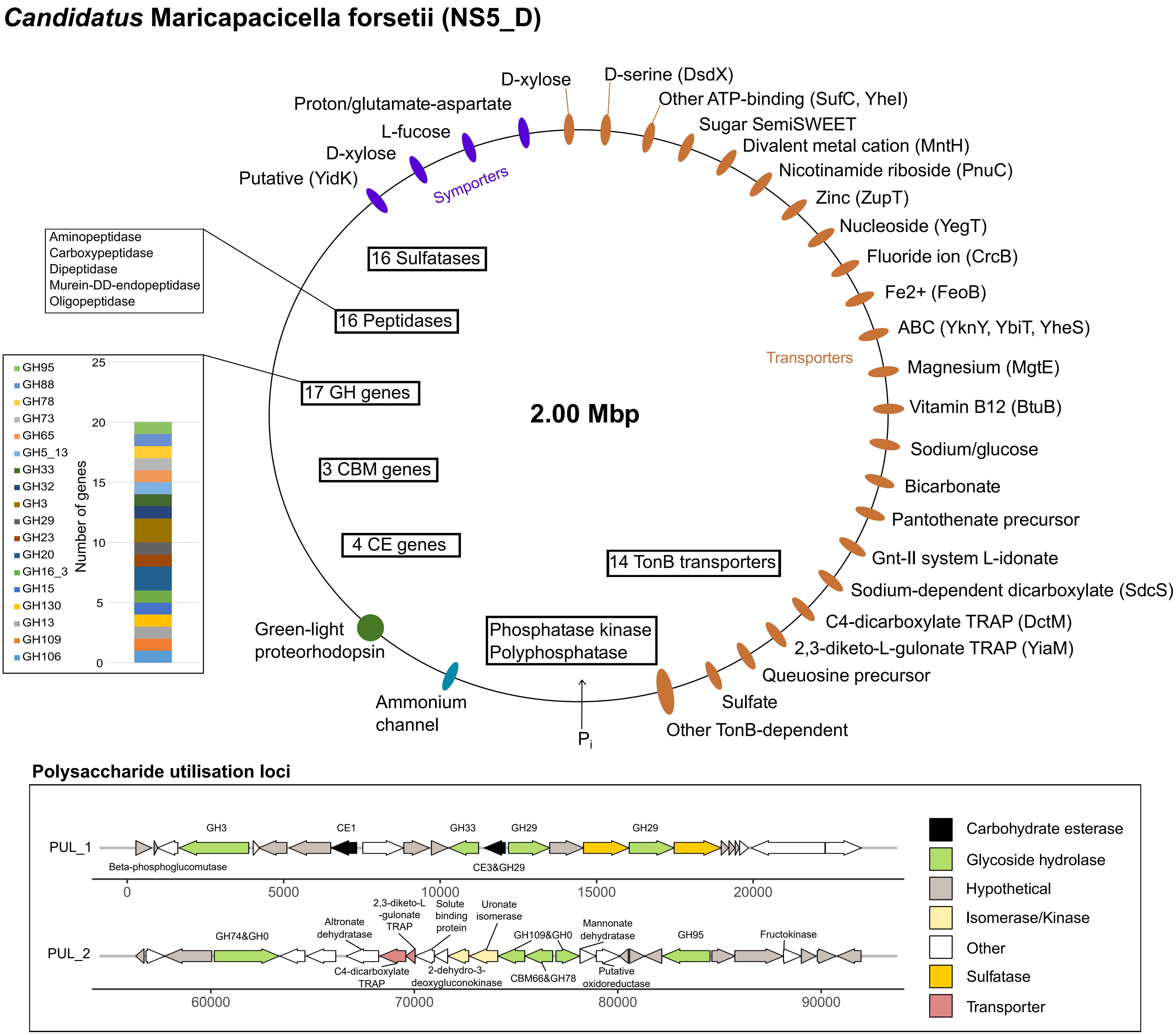

Supplementary Figure S24. Summary schematic of the metabolism of type species *Candidatus* Maricapacicella forsetii

Candidatus Arcticimaribacter forsetii (NS5_F)

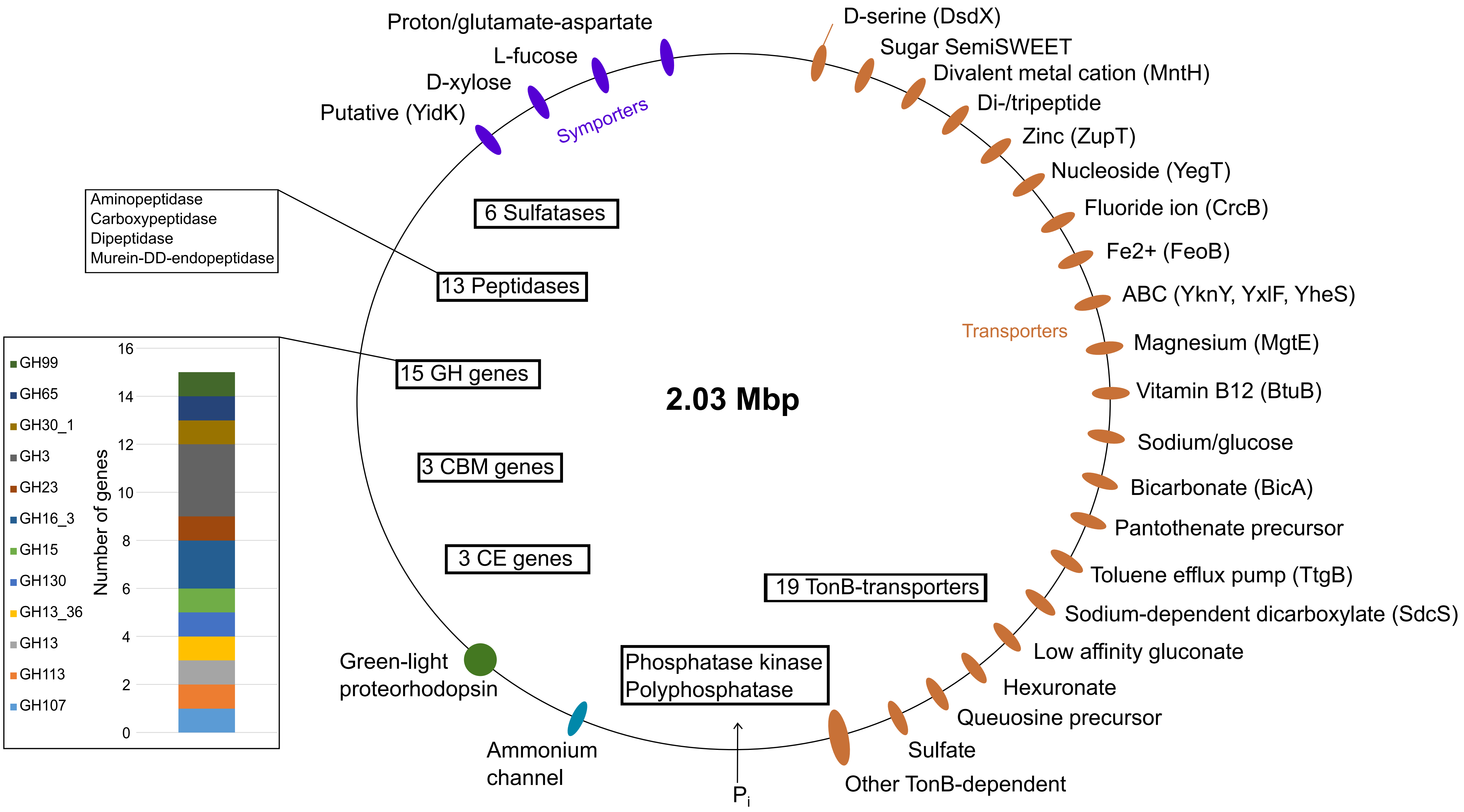

Polysaccharide utilisation loci

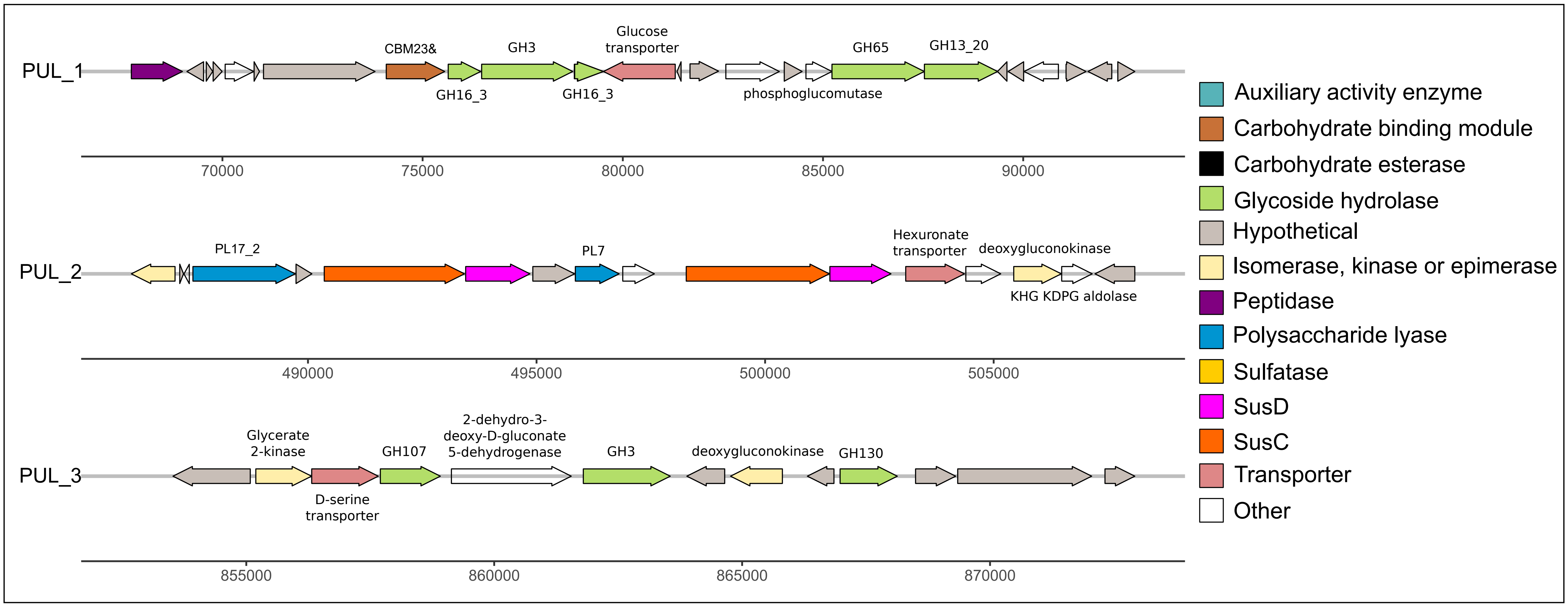

Supplementary Figure S25. Summary schematic of the metabolism of type species *Candidatus* Arcticimaribacter forsetii
